# Supplementary material for: Shared functional specialization in transformer-based language models and the human brain
Source: Nat Commun. 2024 Jun 29;15:5523. doi: 10.1038/s41467-024-49173-5 (PMC11217339; doi:10.1038/s41467-024-49173-5)
Supplement: Supplementary file 1 — Supplementary Information [file 41467_2024_49173_MOESM1_ESM.pdf]

Supplementary Information

Shared functional specialization in transformer-based language models and the human brain

Sreejan Kumar[1,*], Theodore R. Sumers[2,*], Takateru Yamakoshi[3], Ariel Goldstein[1,4], Uri Hasson[1,4], Kenneth A. Norman[1,4], Thomas L. Griffiths[2,4], Robert D. Hawkins[1,4], Samuel A. Nastase[1]

[1]Princeton Neuroscience Institute, Princeton University, Princeton, NJ, 08540, USA
[2]Department of Computer Science, Princeton University, Princeton, NJ 08540, USA
[3]Faculty of Medicine, The University of Tokyo, Bunkyo-ku, Tokyo 113-0033, Japan
[4]Department of Psychology, Princeton University, Princeton, NJ 08540, USA

*Equal contribution

Correspondence: sreejank@princeton.edu, sumers@princeton.edu, snastase@princeton.edu

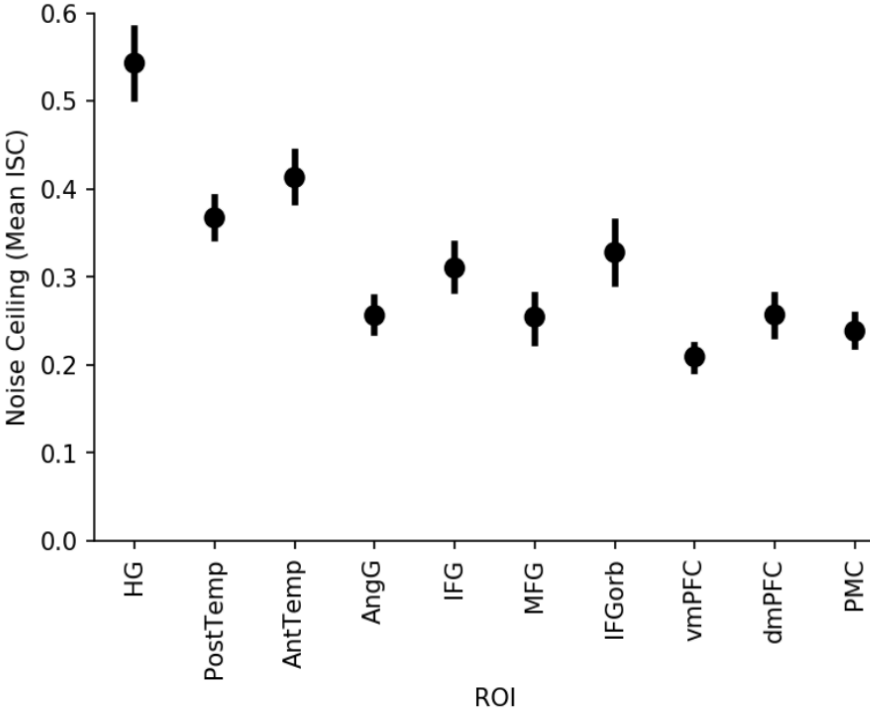

Figure S1. Noise ceilings for each language ROI as measured by intersubject correlation (ISC) analysis (Nastase et al., 2019; cf. Fig. 2). We evaluate the performance of the encoding models by computing the correlation between predicted and actual parcel time series for left-out test segments of each stimulus. To construct a noise ceiling, for each parcel we compute the correlation between each subject's time series and the average time series of the subjects (i.e. leave-one-out ISC). ISC is computed for test segments of each stimulus to match the inputs to the encoding analysis. Here, we report the mean ISC across subjects for each language ROI; error bars represent 95% bootstrap confidence intervals of the mean across subjects. Figure made using Matplotlib and seaborn.

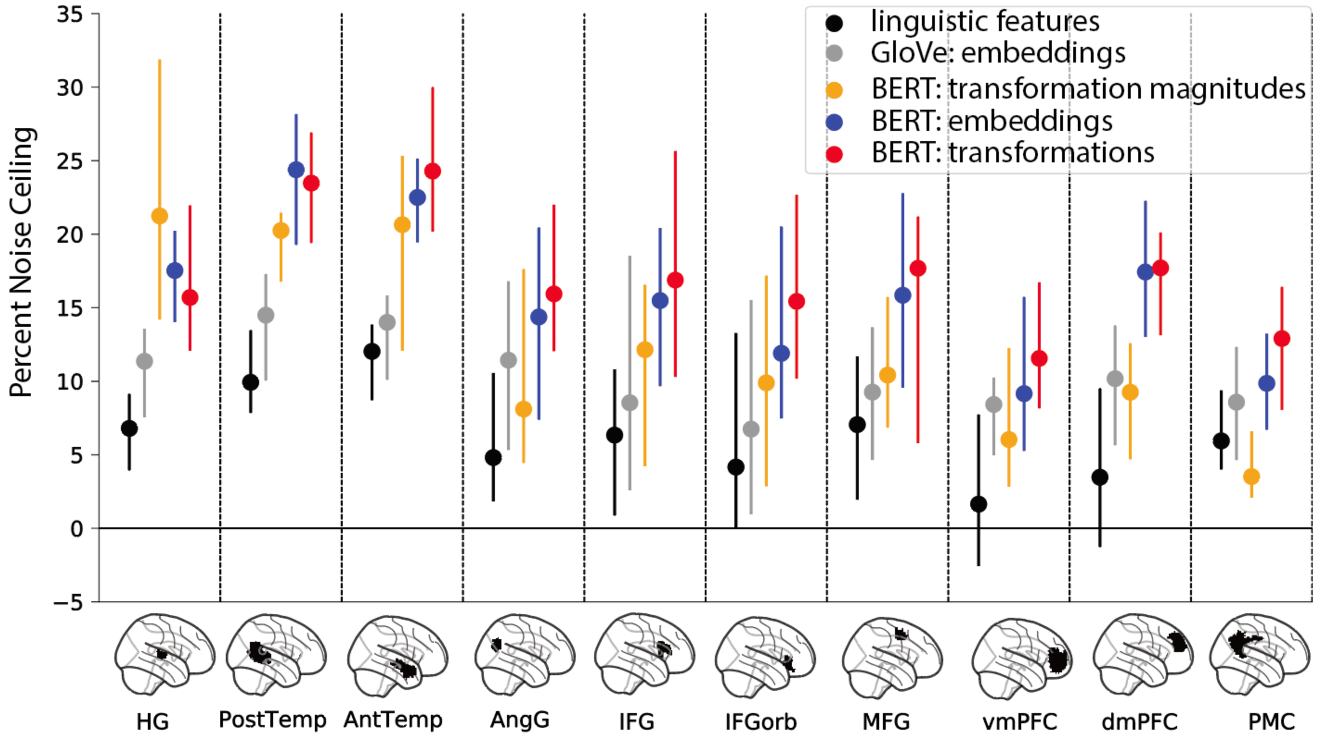

Figure S2. Comparing three classes of language models in right-hemisphere language areas (cf. Fig. 2). Right-hemisphere language areas yield qualitatively similar encoding results to left-hemisphere language areas. Model performance is evaluated in terms of the percent of a noise ceiling estimated using intersubject correlation. Markers indicate median performance and error bars indicate 95% bootstrap confidence intervals. Figure made using Nilearn, Matplotlib, seaborn, and Inkscape.

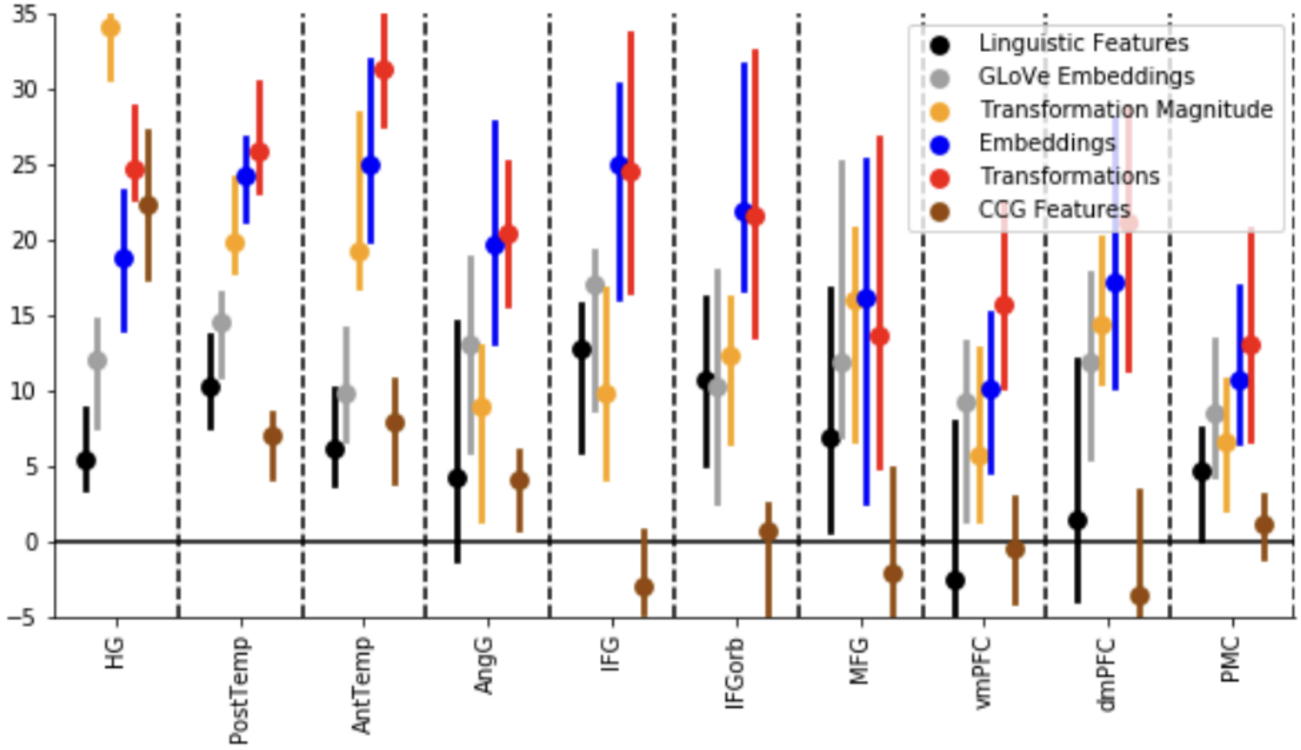

Figure S3. Comparing combinatory categorial grammar (CCG) parser effort to other linguistic features. Note that only the “I Knew You Were Black” story stimulus was used in this comparison. Scalar parsing effort metrics for right-branching, left-branching, and left-branching-with-revealing operations were supplied to the same encoding pipeline used for other types of features: banded ridge regression with three-fold cross-validation. The CCG effort metric (brown) yielded above-zero prediction scores in early auditory cortex, posterior and anterior temporal cortex, as well as angular gyrus, corroborating the results reported by Stanojević and colleagues (2023). In early auditory cortex, the CCG effort metric performed comparably with the embeddings and transformations, but was outperformed by the transformation magnitudes. In all other ROIs, however, the transformer features outperformed CCG effort. Figure made using Matplotlib and seaborn.

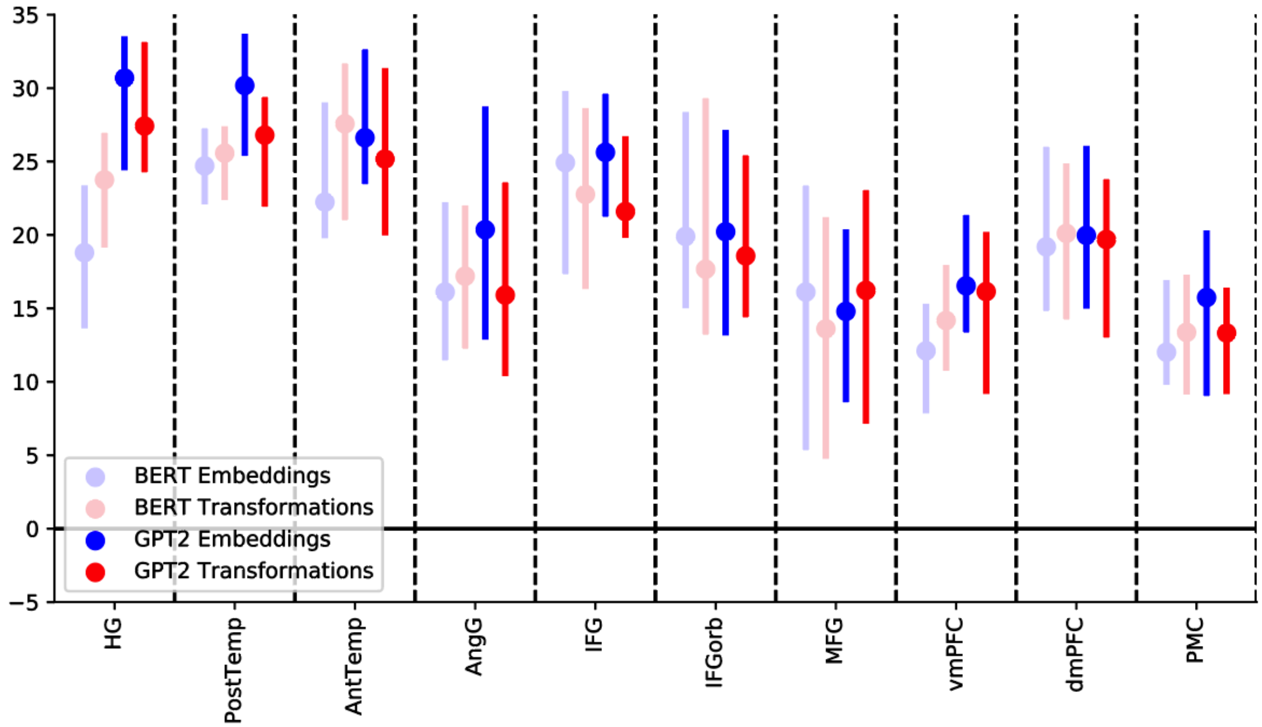

Figure S4. Encoding performance for GPT-2 is comparable to BERT across left-hemisphere language ROIs (cf. Fig. 2). We extracted embeddings (blue) and transformations (red) from GPT-2 and repeated the same encoding analysis as was performed using BERT. Results for BERT are reproduced from Fig. 2 for comparison. Model performance is evaluated in terms of the same noise ceiling used to evaluate BERT. Markers indicate median performance across participants and error bars indicate 95% bootstrap confidence intervals. Figure made using Matplotlib and seaborn.

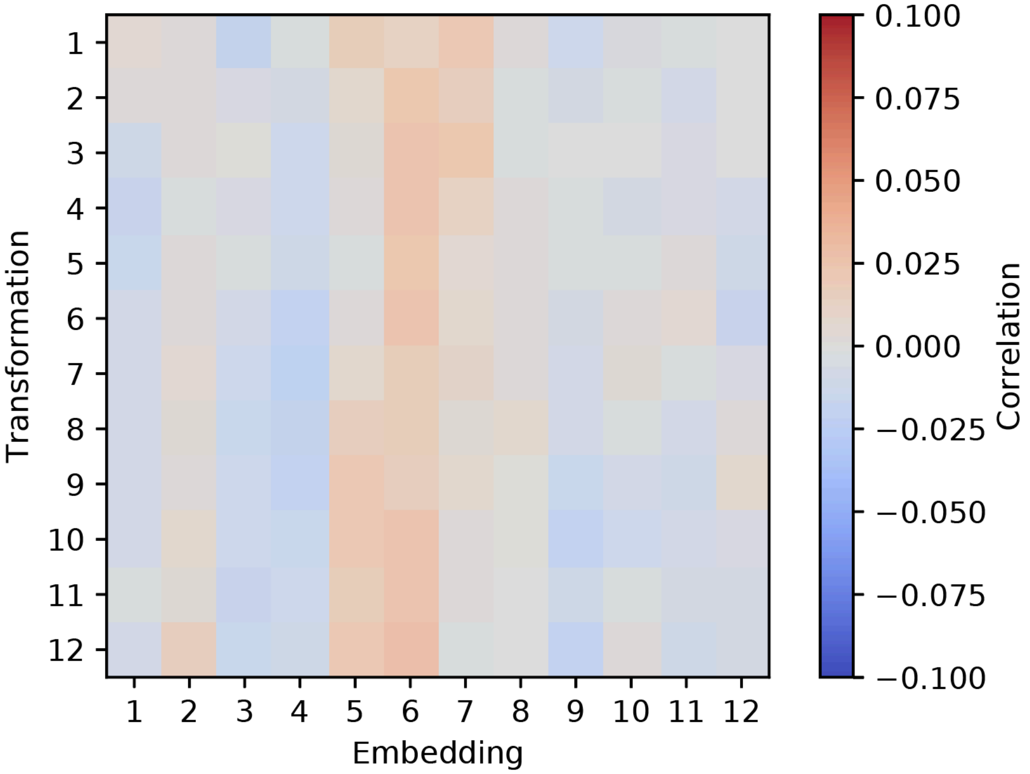

Figure S5. Pairwise correlations between transformations and embeddings across layers. For each TR, we computed the correlation between the transformations and embedding at each pair of layers, then averaged the resulting correlation matrices. As expected, the transformations and embeddings are not strongly correlated, even at adjacent layers. Although the transformations and embeddings have matching dimensionality, they do not share the same feature space. Figure made using Matplotlib, seaborn, and Inkscape.

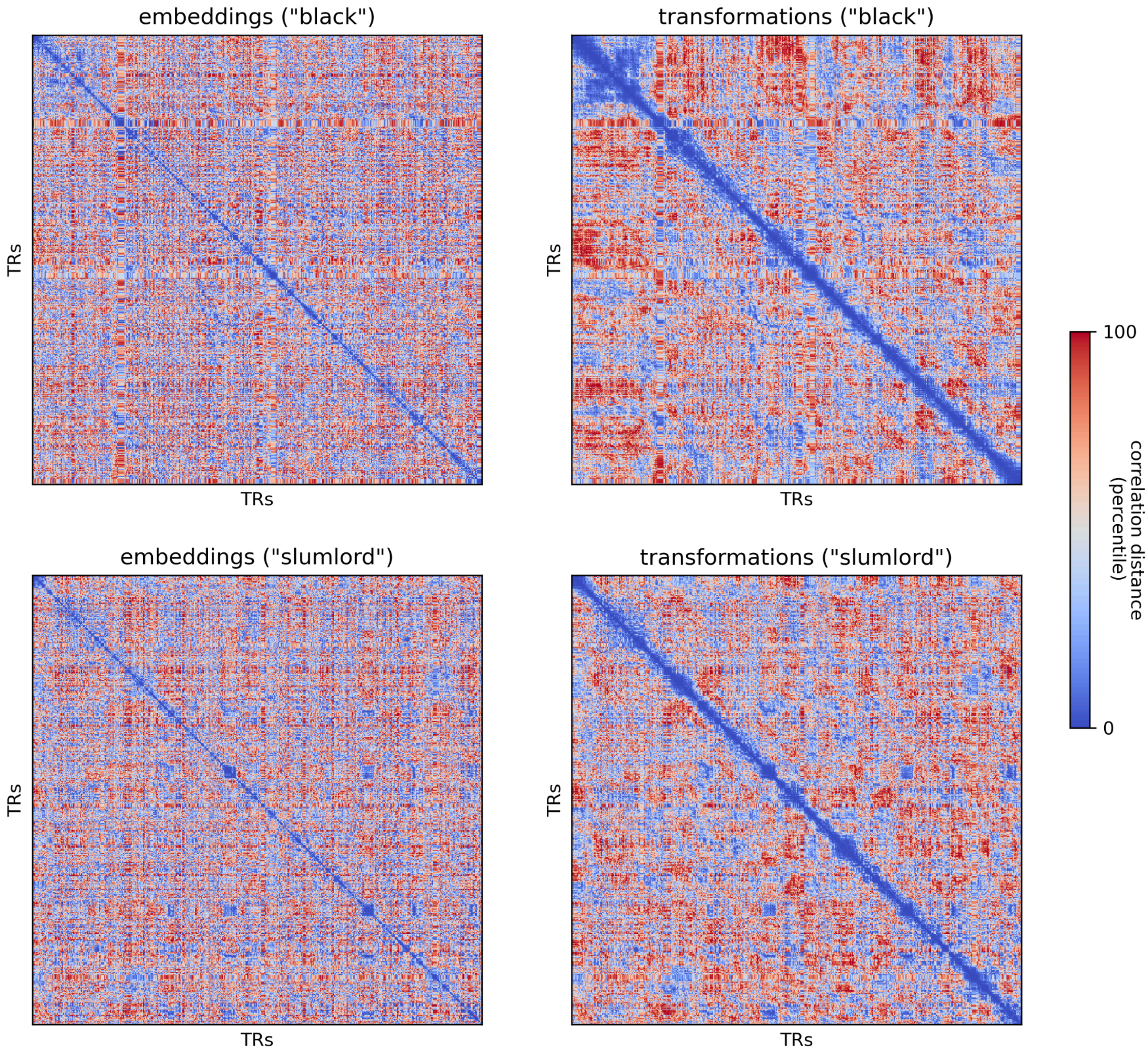

Figure S6. Time-point-by-time-point representational dissimilarity matrices (RDMs) for BERT embeddings (left) and transformations (right) concatenated across all layers. Despite yielding comparable encoding performance across many ROIs, the embeddings and transformations yield different representational geometries. The time-point-by-time-point RDMs are correlated at Spearman r = .768 and r = .815 for the “black” and “slumlord” stimuli, respectively. Relatively high correlations are expected when comparing representational geometries for the entire model (all 12 layers) because the transformations contextually sculpt the embeddings layer-by-layer; the layerwise representational geometries reveal a more obvious difference between embeddings and transformations (Figs. S6, S7). Dissimilarities between the vectors at each TR are measured using correlation distance (1 – Pearson correlation). Dissimilarities are colored according to percentiles. Figure made using Matplotlib and seaborn.

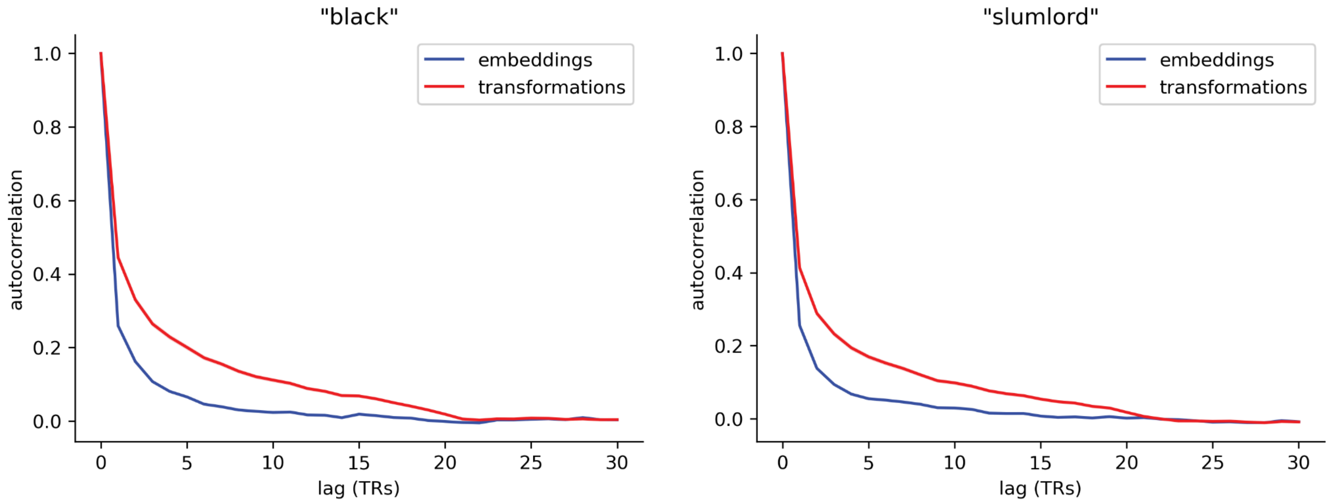

Figure S7. Transformations (red) have higher temporal autocorrelation than embeddings (blue) in both the “black” (left) and “slumlord” (right) story stimuli. We computed the TR-by-TR autocorrelation up to a lag of 30 TRs (45 s) separately for each feature in the embedding and transformation vectors. The blue and red lines indicate the mean temporal autocorrelation across features for embeddings and transformations, respectively. Bootstrap 95% confidence intervals are plotted around the means, but are not visible given the highly consistent temporal autocorrelation functions across features. Figure made using Matplotlib and seaborn.

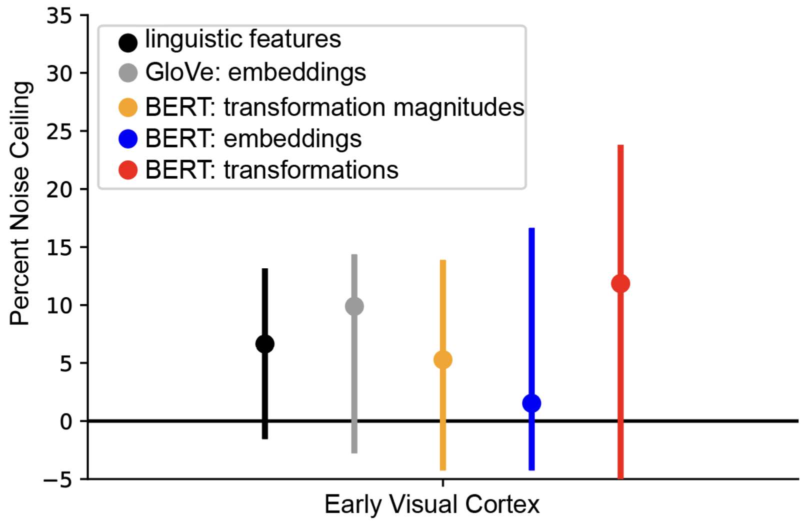

Figure S8. Encoding performance for three classes of language models in an early visual control ROI (cf. Fig. 2). Early visual cortex was anatomically defined as parcels overlapping with Brodmann Area 17 (primary visual cortex). As expected, none of these language features yield statistically significant prediction performance in early visual parcels. Model performance is evaluated in terms of the percent of a noise ceiling estimated using intersubject correlation. Markers indicate median performance and error bars indicate 95% bootstrap confidence intervals. Figure made using Matplotlib, seaborn, and Inkscape.

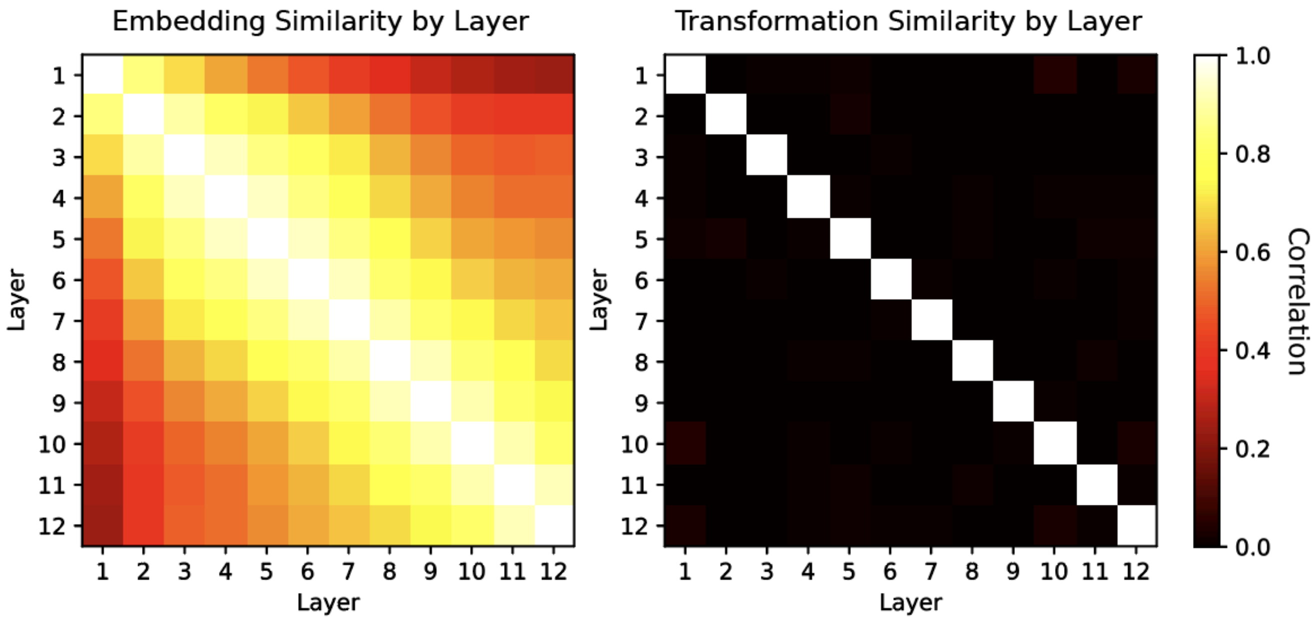

Figure S9. Correlation between embeddings across layers (left) and transformations across layers (right). Each word of the story stimulus was propagated through the model and the transformations and embeddings were extracted at each layer. We averaged transformations and embeddings for each word within a TR (see "Generating Transformer features" in Methods). We computed the pairwise correlation of the transformations and embeddings across the 12 layers for each TR (each TR yields a 12 × 12 correlation matrix), then averaged the resulting correlation matrices across TRs. Embeddings are much more similar across layers because the residual connections allow the embeddings to accumulate information (or remain unchanged) across layers. On the other hand, transformations capture layer-by-layer "updates" to the embedding and are largely uncorrelated across layers. Figure made using Matplotlib and seaborn.

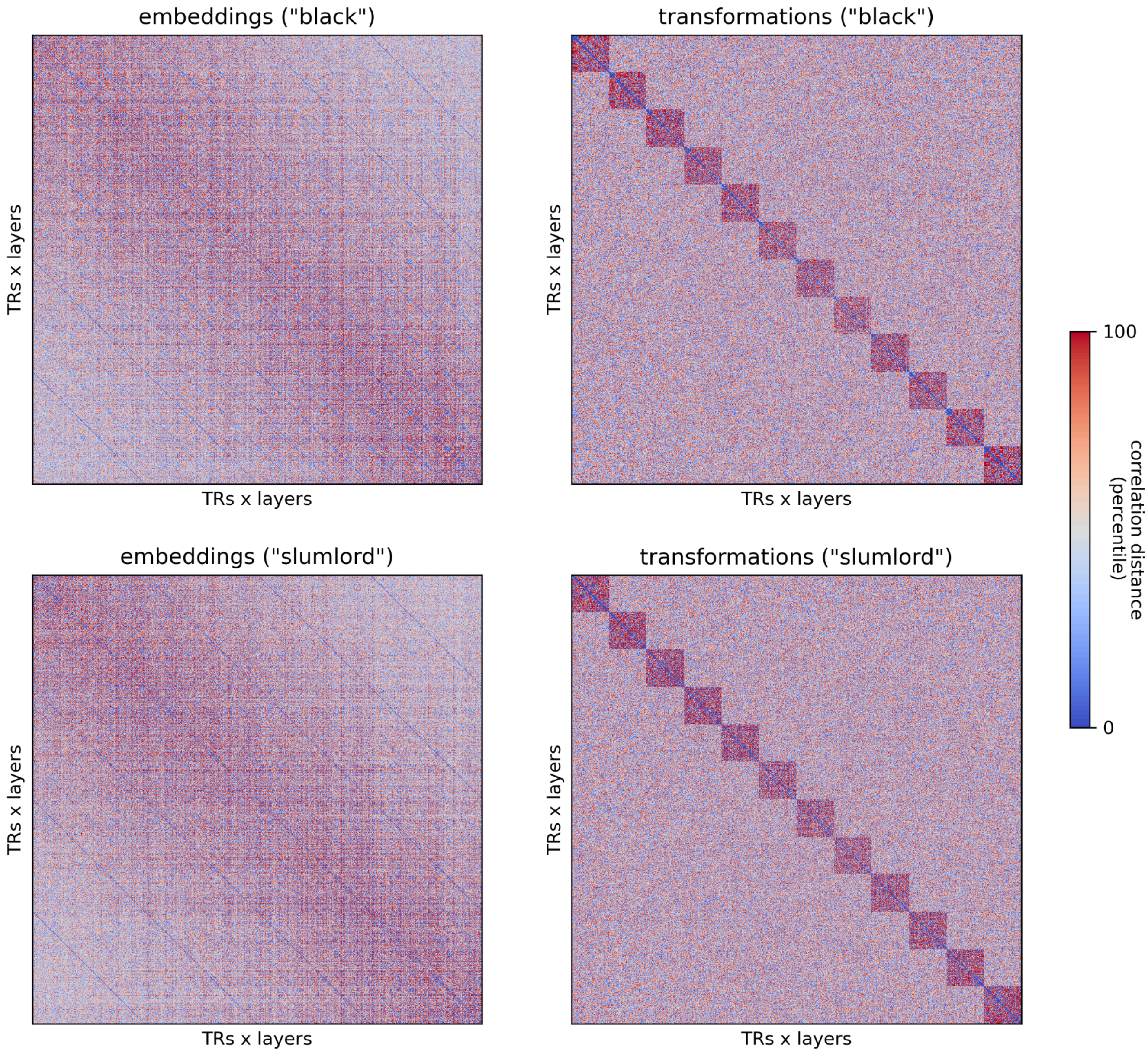

Figure S10. Time-point-by-time-point RDMs for embeddings (left) and transformations (right) within and across each layer of BERT. To compute the RDMs, we first split the embeddings and transformations by layer, then stacked these layers along the time axis; for example, the 12 diagonal blocks in the transformation RDMs (right) correspond to time-point-by-time-point RDMs at each layer. Cross-layer dissimilarities assume that the 768 features at each layer are shared across layers, and this is not strictly true by design. However, the embedding vectors are much more similar across layers than the transformation vectors. Overall, the RDMs for embeddings and transformations are highly dissimilar; Spearman r = .080 and .083 for the “black” and “slumlord” story stimuli, respectively. Dissimilarities between the vectors at each TR are measured using correlation distance (1 – Pearson correlation). Dissimilarities are colored according to percentiles. Figure made using Matplotlib and seaborn.

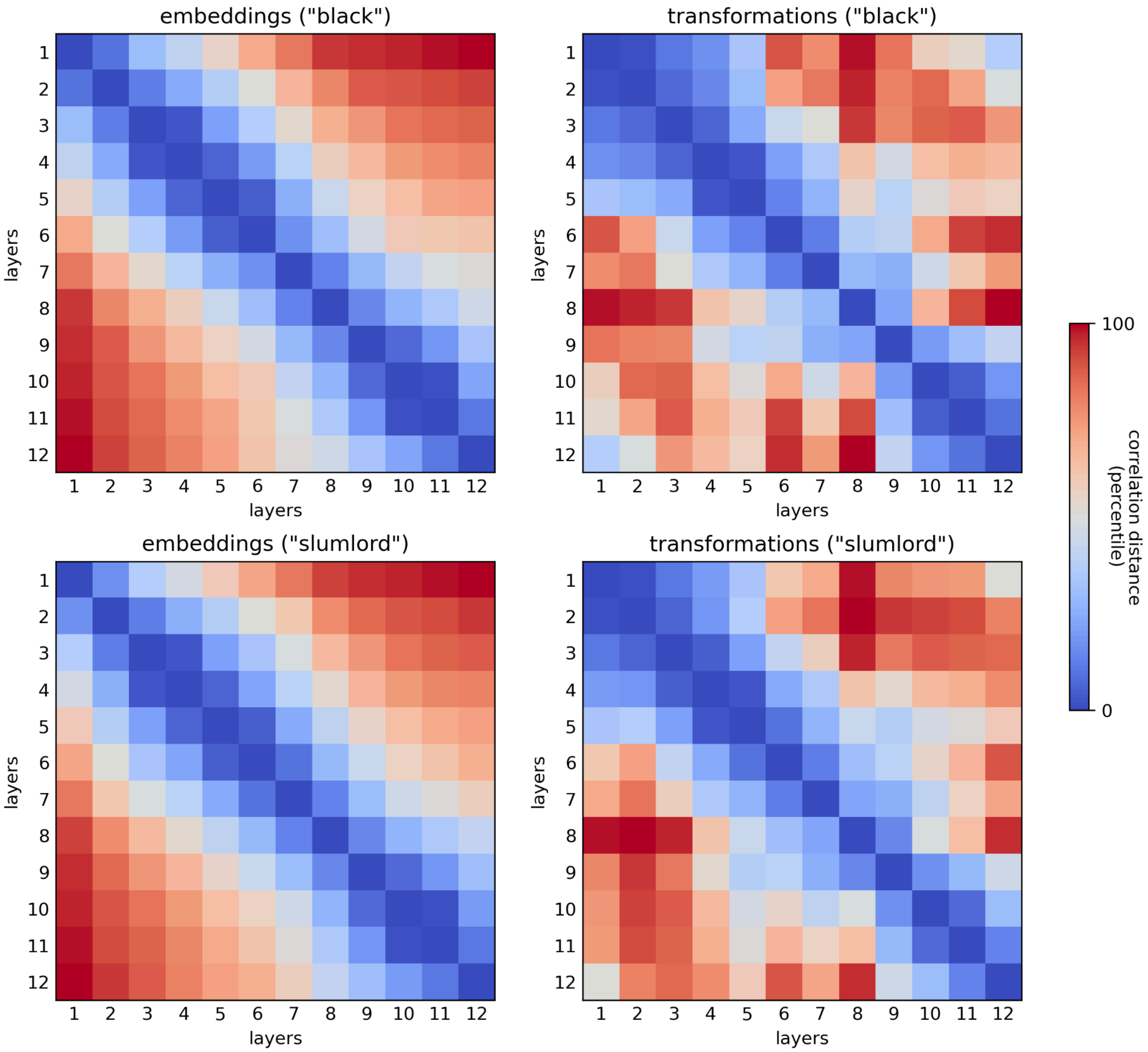

Figure S11. Second-order layer-by-layer representational geometry of the time-point-by-time-point RDMs for embeddings (left) and transformations (right). We first computed a time-point-by-time-point RDM for each of the 12 layers. We computed the pairwise dissimilarity of these time-point RDMs across all 12 layers, resulting in a 12-by-12 RDM. The layerwise embedding RDMs (left) demonstrate that the layerwise representational geometries evolve sequentially across layers; i.e. the representational geometry at each layer is most similar to neighboring layers and most distinct from the most distant layers. The layerwise transformation RDMs (right), on the other hand, demonstrate that the layerwise representational geometries for transformations are not as sequentially organized; for example, early-layer transformations are more similar to particular late-layer transformations than they are to particular intermediate-layer transformations. The second-order layer-by-layer RDMs are correlated at Spearman r = .750 and r = .848 for the “black” and “slumlord” stimuli, respectively. Second-order dissimilarities between the time-point-by-time-point RDMs are measured using correlation distance (1 – Pearson correlation). Dissimilarities are colored according to percentiles. Figure made using Matplotlib and seaborn.

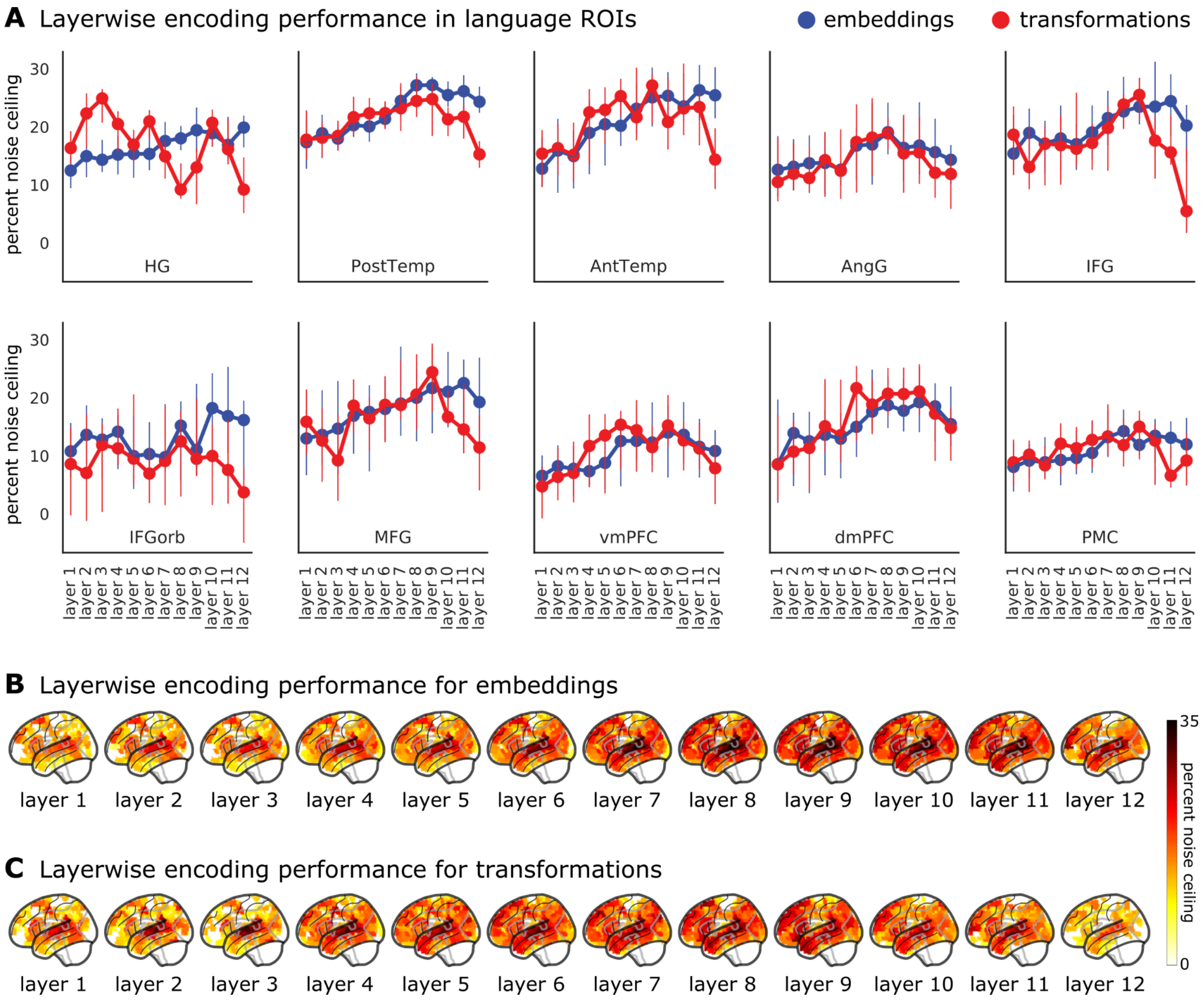

Figure S12. Layerwise performance of embeddings and transformations. (**A**) Layerwise model performance for embeddings (blue) and transformations (red) in ten left-hemisphere language ROIs (see Fig. S13 for right-hemisphere language areas). Model performance for embeddings tends to increase monotonically across layers, whereas performance for embeddings tends to peak for intermediate layers. Markers indicate median performance and error bars indicate 95% bootstrap confidence intervals. (**B**) Model performance for each layer of embeddings and (**C**) transformations across all cortical parcels. Cortical maps are thresholded to display only parcels with statistically significant model performance (nonparametric bootstrap hypothesis test; FDR controlled at $p < .05$). Model performance is evaluated in terms of the percent of a noise ceiling estimated using intersubject correlation. Figure made using Nilearn, Matplotlib, seaborn, and Inkscape.

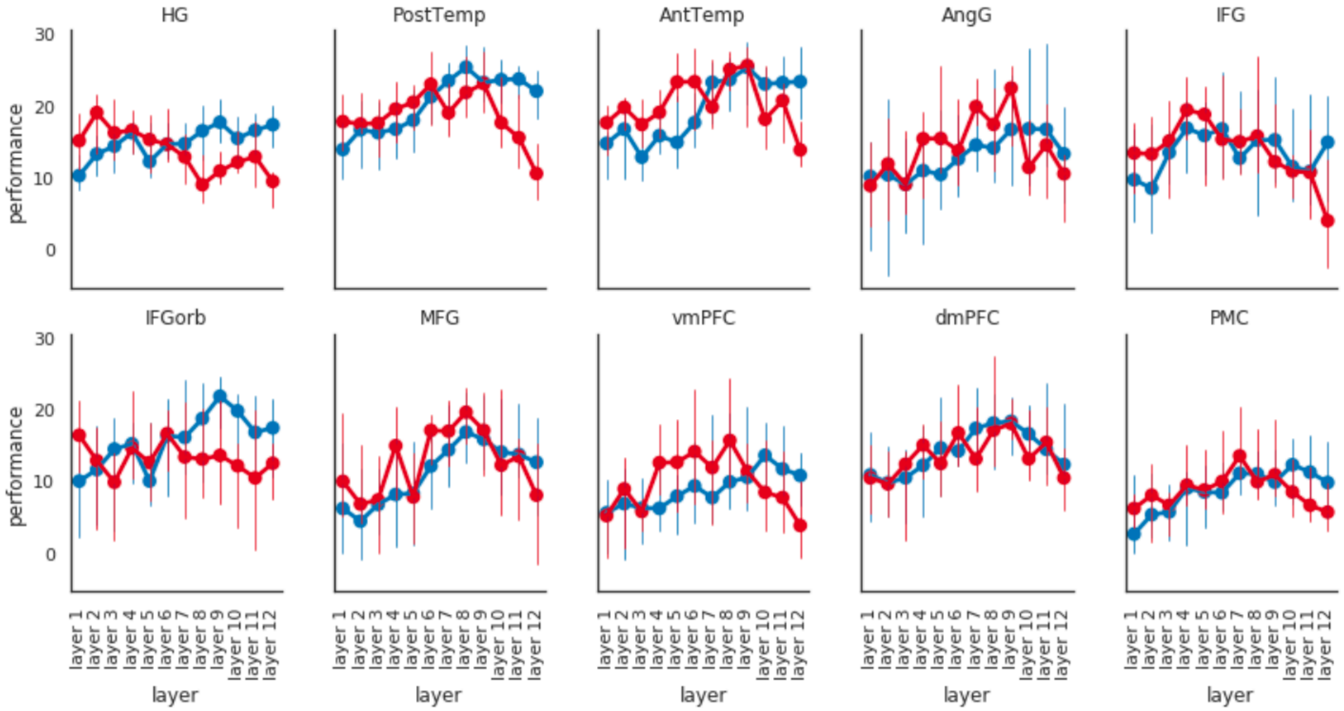

Figure S13. Layerwise encoding performance for embeddings (blue) and transformations (red) in right-hemisphere language areas (cf. Fig. S12A). Model performance is evaluated in terms of the percent of a noise ceiling estimated using intersubject correlation. Markers indicate median performance and error bars indicate 95% bootstrap confidence intervals. Figure made using Matplotlib and seaborn.

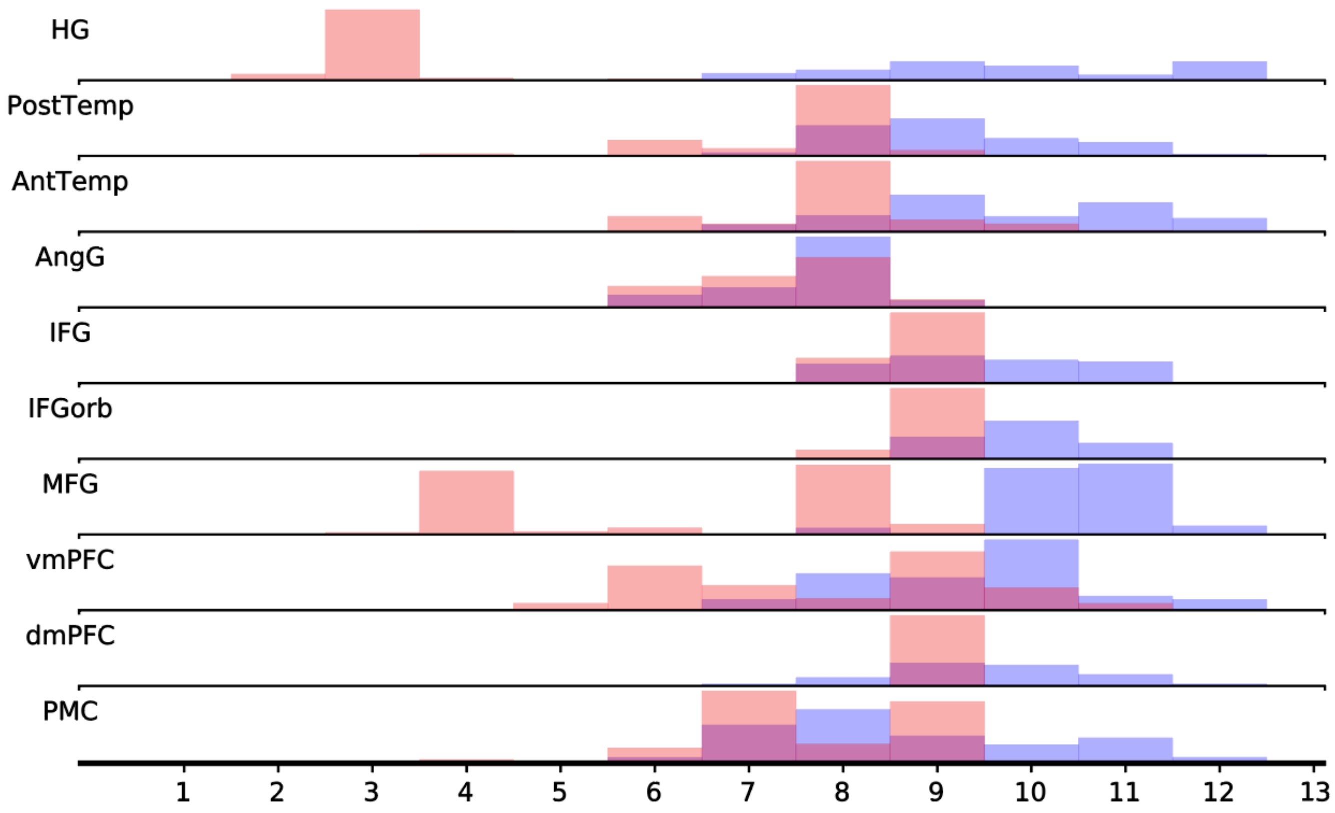

Figure S14. To examine the relative specificity of these layer preferences, we examined the entropy of the softmax distribution across layer performances (Tenney et al., 2019) for language ROIs, where higher entropy corresponds to a relatively uniform distribution while lower entropy corresponds to a relatively concentrated distribution. We found that performance scores for transformations were significantly more layer-specific than embeddings overall ($p < .05$ for all language ROIs; Table S3) where performance scores for embeddings were relatively diffuse across final layers. Softmax distribution of transformation and embedding performances across different layers. The distribution of layer preferences for embeddings is more diffuse across layers than that of the transformations. To quantify this layer specificity, we quantified the entropy of these softmax distributions and found that the entropy of the embedding distribution was significantly higher than that of the transformation distribution in all of the ROIs (Table S3). Figure made using Matplotlib and seaborn.

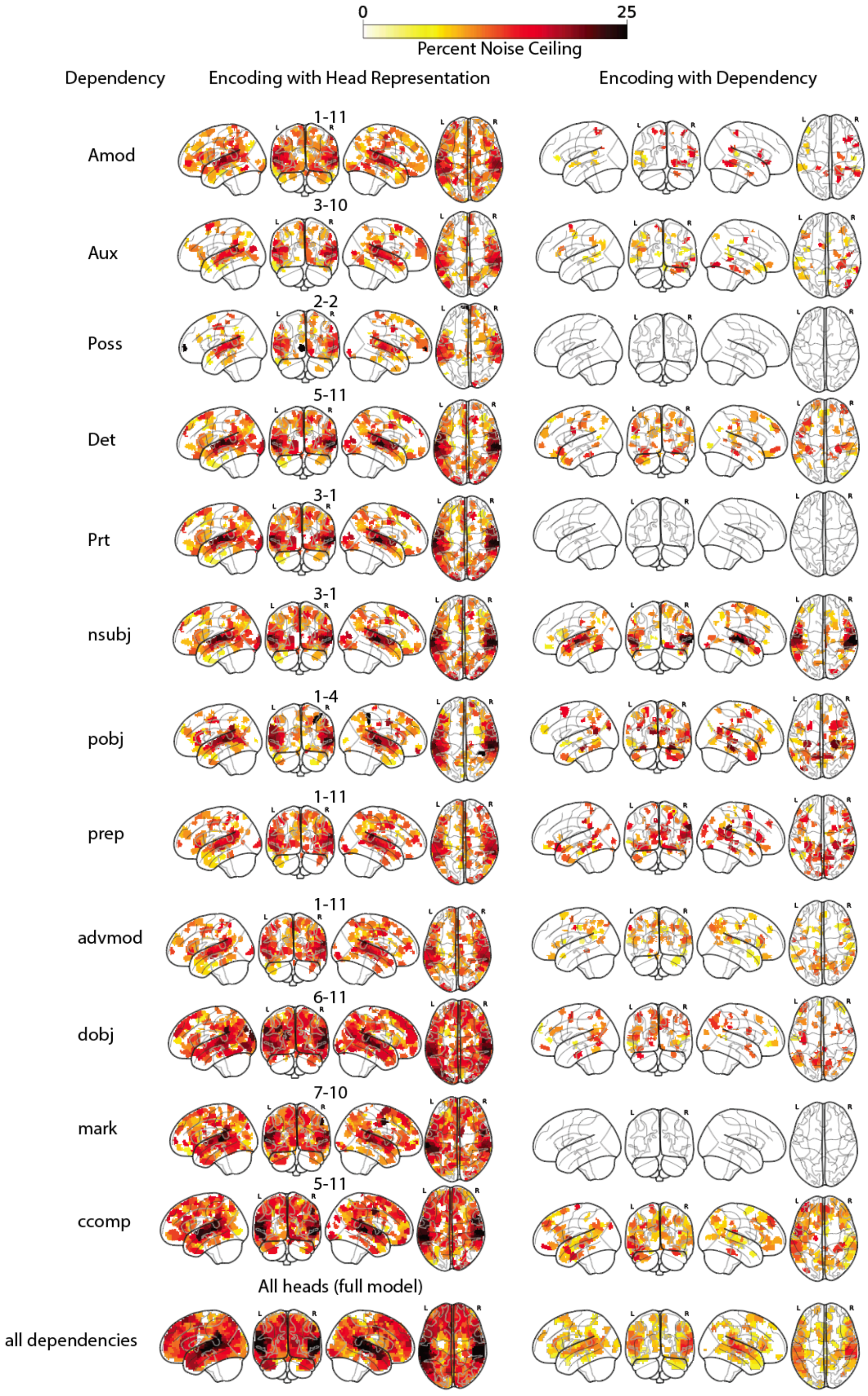

Figure S15. Comparison between encoding performance using the headwise transformation that best decodes a particular classical linguistic dependency (left) and the indicator variable for the linguistic dependency itself (right). The best-performing head is labeled (layer-head) for each dependency. Cortical maps are thresholded to display only parcels with statistically significant model performance (nonparametric bootstrap hypothesis test;

FDR controlled at $p < .05$). Model performance is evaluated in terms of the percent of a noise ceiling estimated using intersubject correlation. Note that for the poss, prt, and mark dependencies, no significant parcels were found. Figure made using Nilearn, Matplotlib, seaborn, and Inkscape.

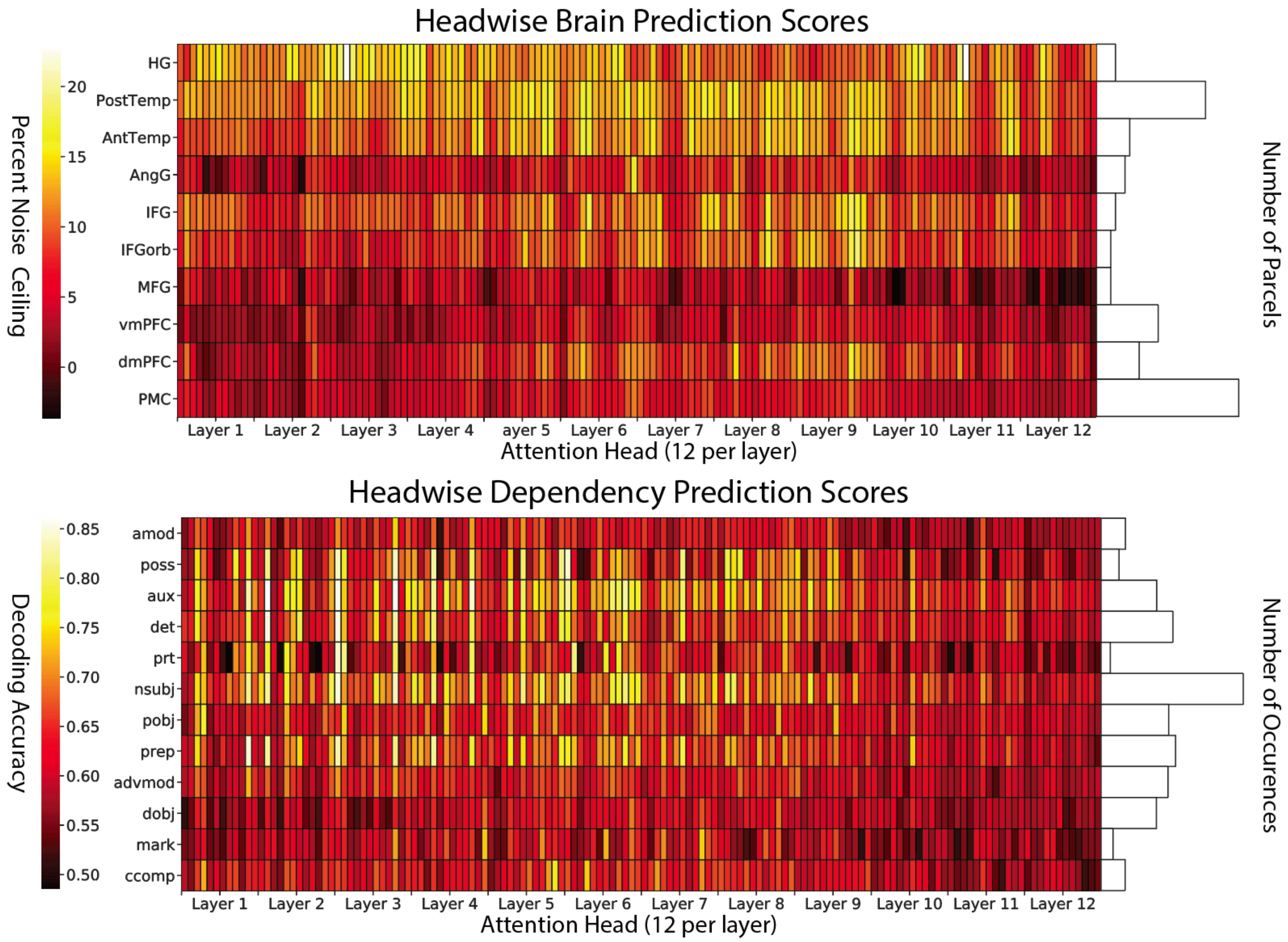

Figure S16. Headwise brain prediction scores (top) and headwise dependency prediction scores (bottom). At top, the headwise brain prediction scores are shown for the 12 layers of BERT (x-axis) and 10 language ROIs (y-axis). There are 12 heads per layer in BERT. The histogram at right reflects the number of parcels in each language ROI. Brain prediction scores reflect cross-validated encoding model performance evaluated in terms of the percent of a noise ceiling estimated using intersubject correlation. At bottom, the headwise dependency prediction scores are shown for the 12 layers of BERT (x-axis) and 12 classical linguistic dependencies. Dependency prediction scores reflect the classification accuracy of a cross-validated logistic regression model trained to predict the occurrence of a given linguistic dependency at each time point from the 64-dimensional transformation vector for a given attention head. The histogram at right reflects the number of occurrences of each linguistic dependency across both story stimuli (Table S6). Figure made using Matplotlib, seaborn, and Inkscape.

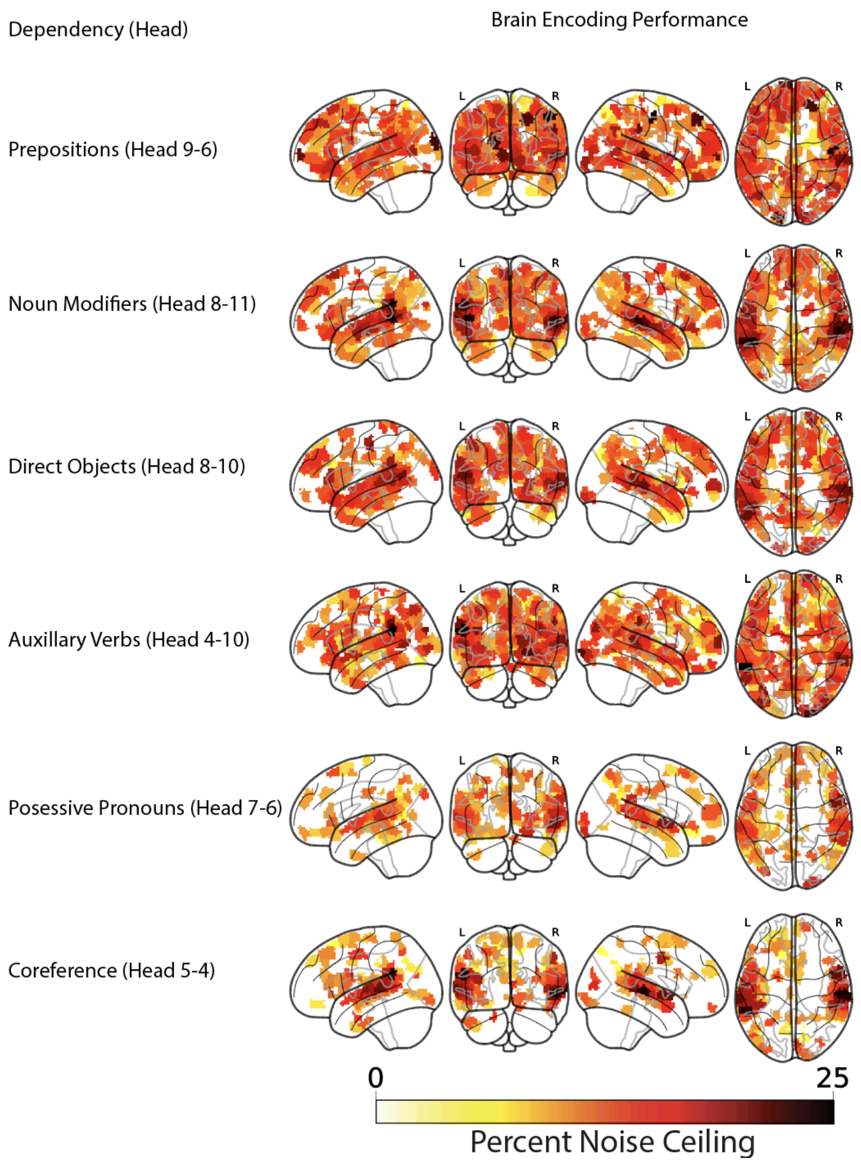

Figure S17. Encoding performance for heads previously highlighted in the literature for functional specialization (Clark et al., 2019). Heads are labeled according to the linguistic phenomenon for which they are specialized as well as their index in BERT (layer-head). Model performance is evaluated in terms of the percent of a noise ceiling estimated using intersubject correlation. Figure made using Nilearn, Matplotlib, seaborn, and Inkscape.

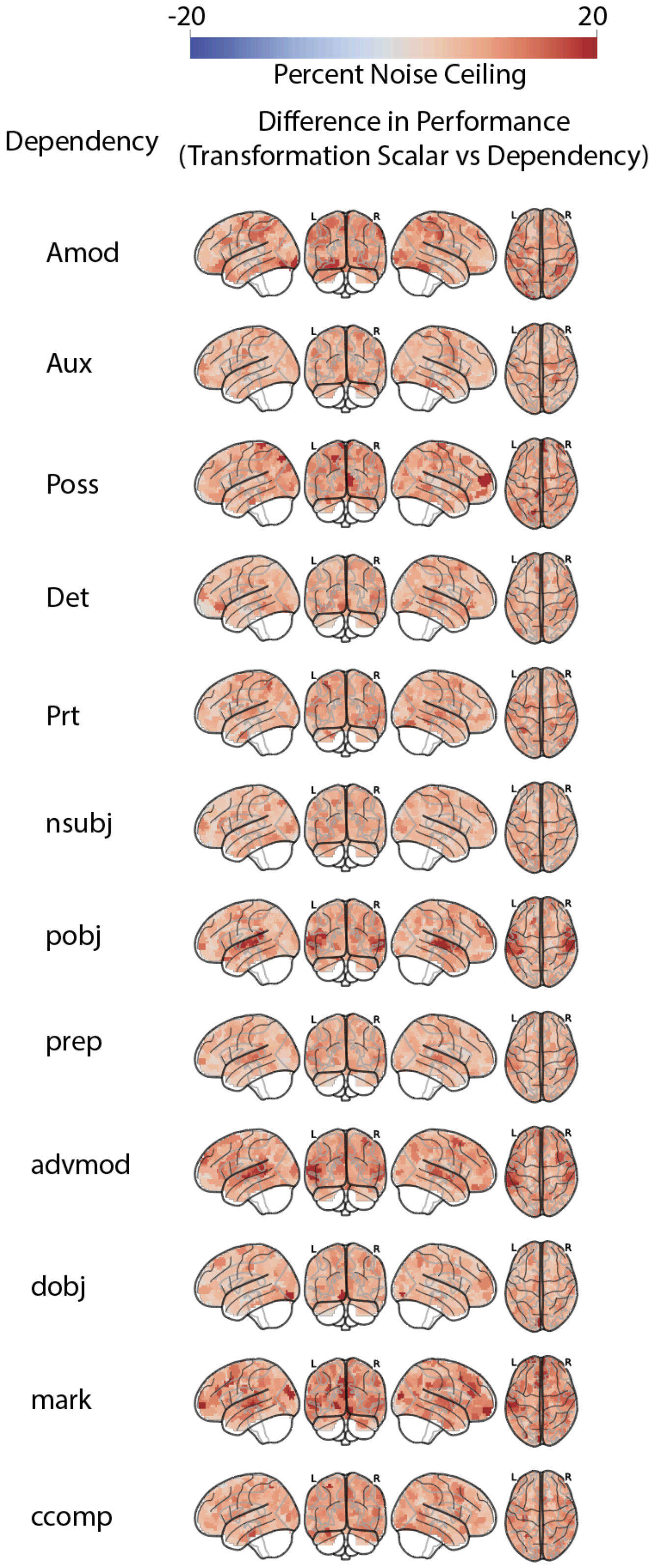

Figure S18. Difference in encoding performance between headwise transformations reduced to a one-dimensional scalar vector and the dependency indicator variable (unthresholded). We reduced the 64-dimensional transformation vector for the head that best decodes a particular dependency down to a single scalar by computing a weighted sum across dimensions based on the weights estimated from the logistic

regression used to decode that dependency (Fig. S13). Despite the matched dimensionality, warm colors indicate that the head-specific transformation values at each TR still outperform the corresponding classical dependency indicator variable in a cross-validated encoding analysis. Figure made using Nilearn, Matplotlib, seaborn, and Inkscape.

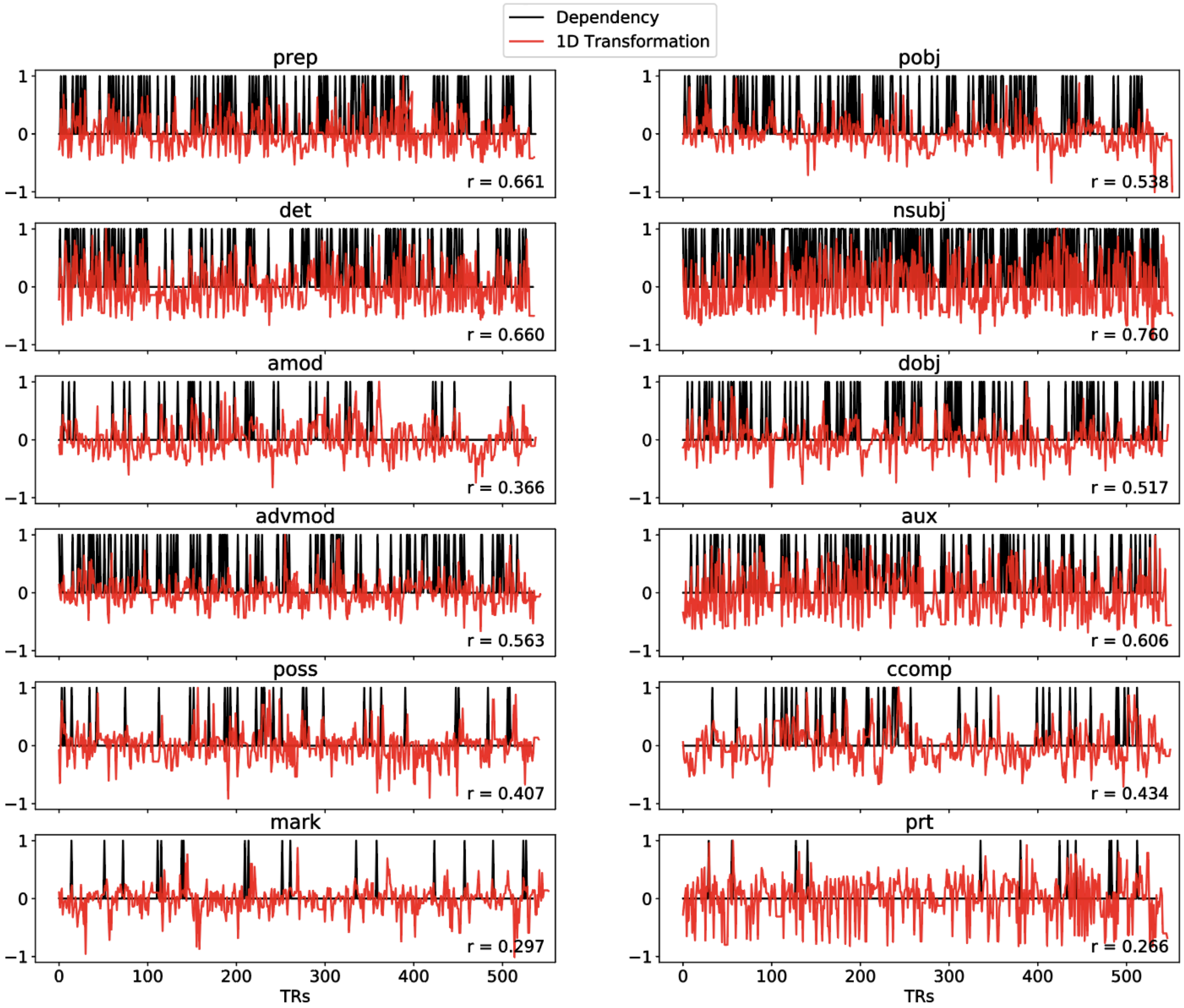

Figure S19. Time series of the one-dimensional transformation that best predicts a given linguistic dependency (red) and the indicator time series of the dependency itself (black) for the full 534 TRS in the "I Knew You Were Black" story stimulus. We first obtained the 64-dimensional transformation vector (corresponding to an attention head) that best predicted a given classical linguistic dependency, assessed using logistic regression (see the "Decoding dependency relations" section of "Methods"). To better match the dimensionality of the transformation vector and the one-dimensional linguistic dependency indicator, we computed a weighted sum of the coefficients estimated using logistic regression. This reduces the 64-dimensional transformation vector a single scalar value (Fig. S15). We then plotted how this one-dimensional transformation fluctuates over time (red) with respect to the linguistic dependency indicator (black). We mean-centered and normalized the one-dimensional transformation time series by the maximum absolute value for visualization. The one-dimensional transformation captures a graded, continuous representation of the linguistic dependency with peaks corresponding to occurrences of the linguistic dependency; correlations between the one-dimensional transformation and linguistic dependency time series are plotted at the bottom right of each panel. Figure made using Matplotlib and seaborn.

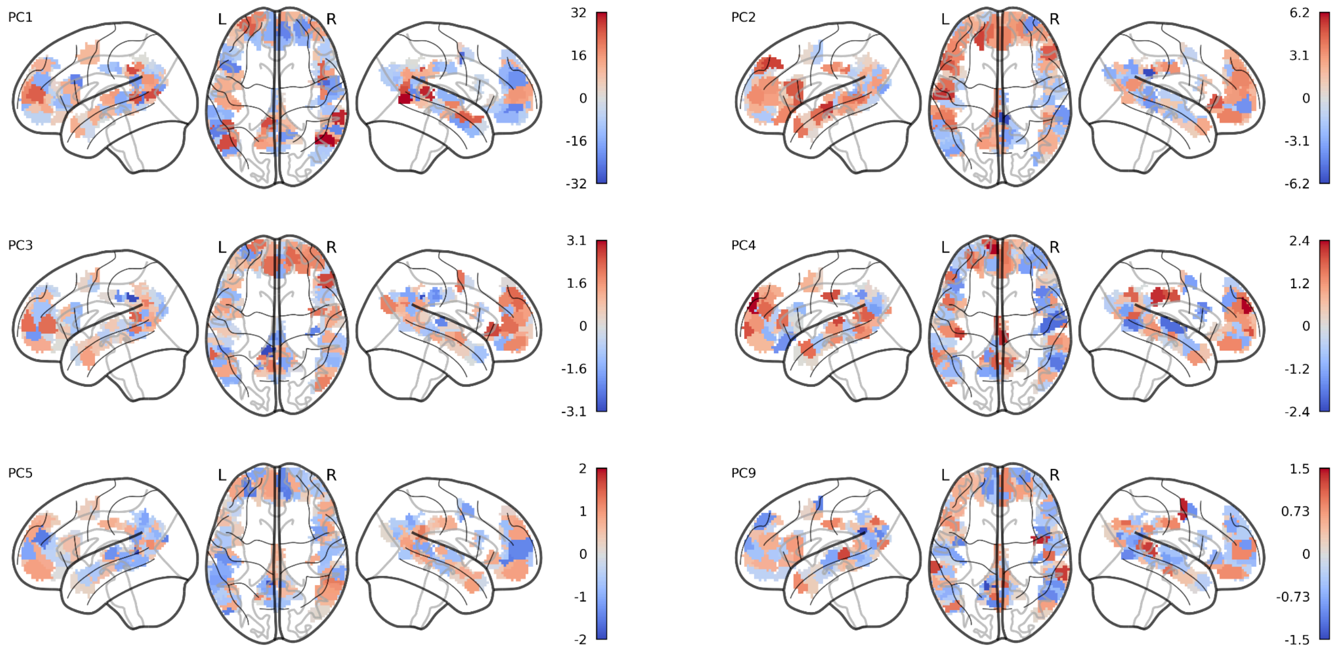

Figure S20. Projection of six PCs summarizing regression coefficients for the transformation-based encoding models (i.e. headwise transformation weights) onto the parcels of the language network (cf. Fig. 4). The PCs are orthogonal and capture non-redundant structure in the transformation weight matrix across the language network. Color bars reflect variance of the transformation weights for each PC (the polarity of each PC is arbitrary). Figure made using Nilearn, Matplotlib, and seaborn.

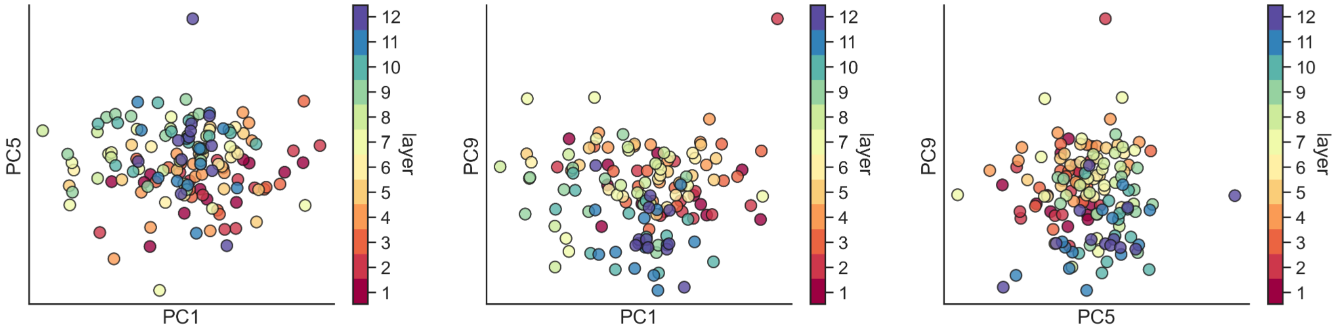

Figure S21. Headwise transformations weights projected into a two-dimensional space summarizing the language network colored according to the layer of each head (cf. Fig. 4D). PCs 9, 5, and 1 were the PCs most correlated with layer assignment with r = .45, .40, and .26, respectively. The PCs are orthogonal and capture non-redundant structure in the transformation weight matrix across the language network. Each PC can be projected back onto the cortical language network (Fig. S18). Each point in a scatter plot corresponds to one of the 144 attention heads. Figure made using Matplotlib and seaborn.

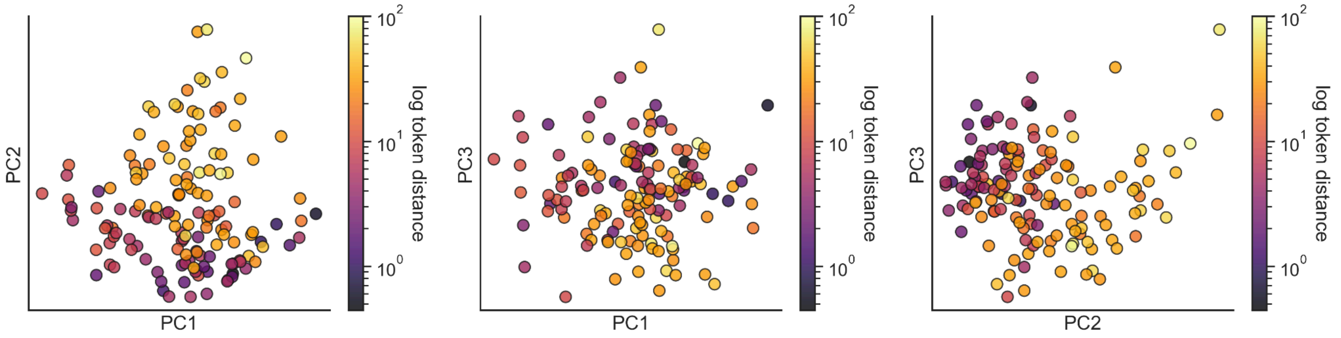

Figure S22. Headwise transformation weights projected into a two-dimensional space summarizing the language network colored according to the backward attention distance of each head (cf. Fig. 4E). PCs 2, 1, and 3 were the PCs most correlated with backward attention distance with r = .65, .20, .19, respectively. The PCs are orthogonal and capture non-redundant structure in the transformation weight matrix across the language network. Each PC can be projected back onto the cortical language network (Fig. S20). Each point in a scatter plot corresponds to one of the 144 attention heads. Figure made using Matplotlib and seaborn.

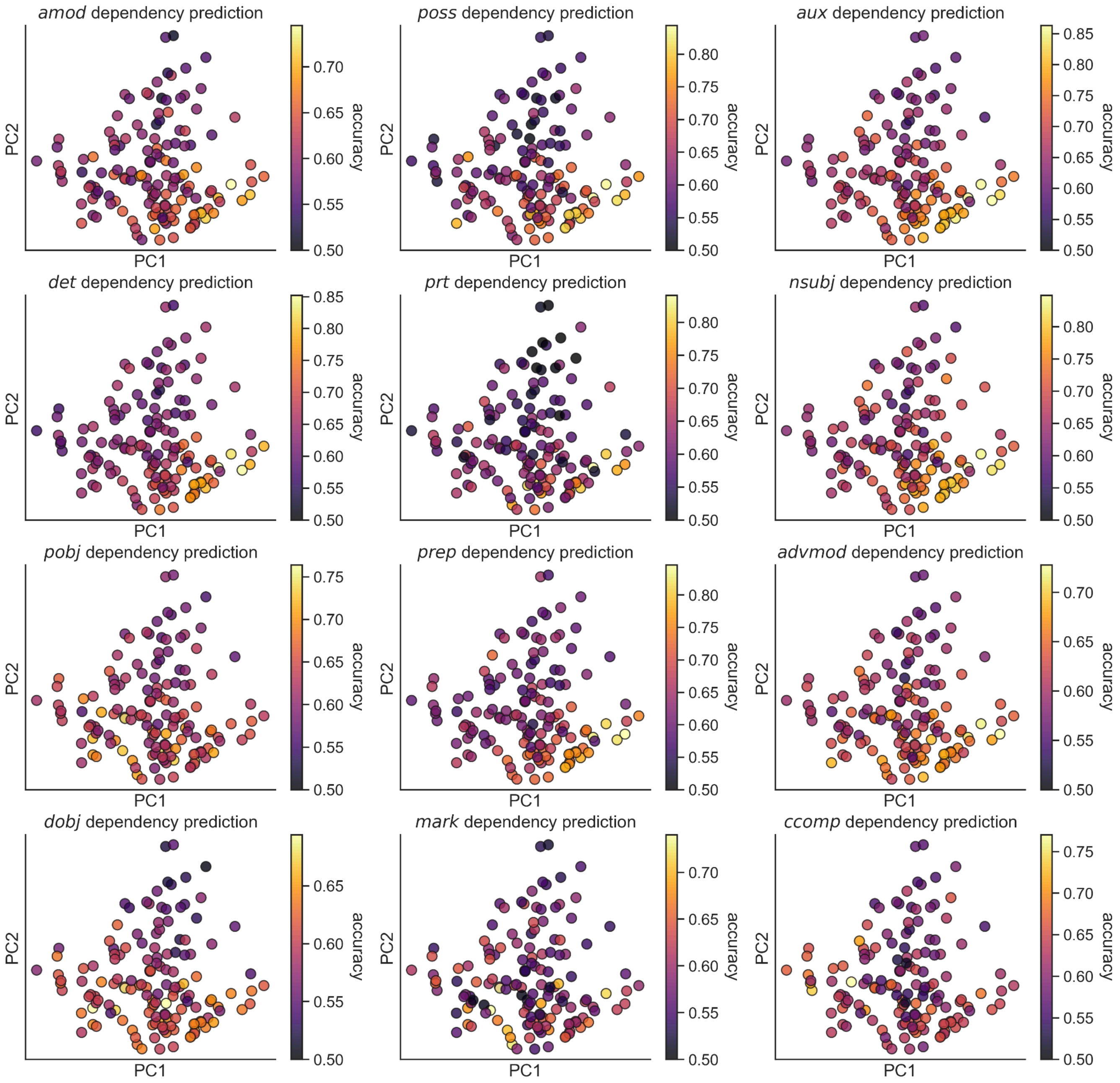

Figure S23. Headwise transformation weights projected into a two-dimensional space summarizing the language network colored according to each head's dependency prediction score for each classical linguistic dependency (cf. Fig. 4). Dependency prediction scores reflect the classification accuracy of a cross-validated logistic regression model trained to predict the occurrence of a given linguistic dependency at each TR from the 64-dimensional transformation vector for a given attention head. The PCs are orthogonal and capture non-redundant structure in the transformation weight matrix across the language network. Each PC can be projected back onto the cortical language network (Fig. S18). Each point in a scatter plot corresponds to one of the 144 attention heads. Figure made using Matplotlib and seaborn.

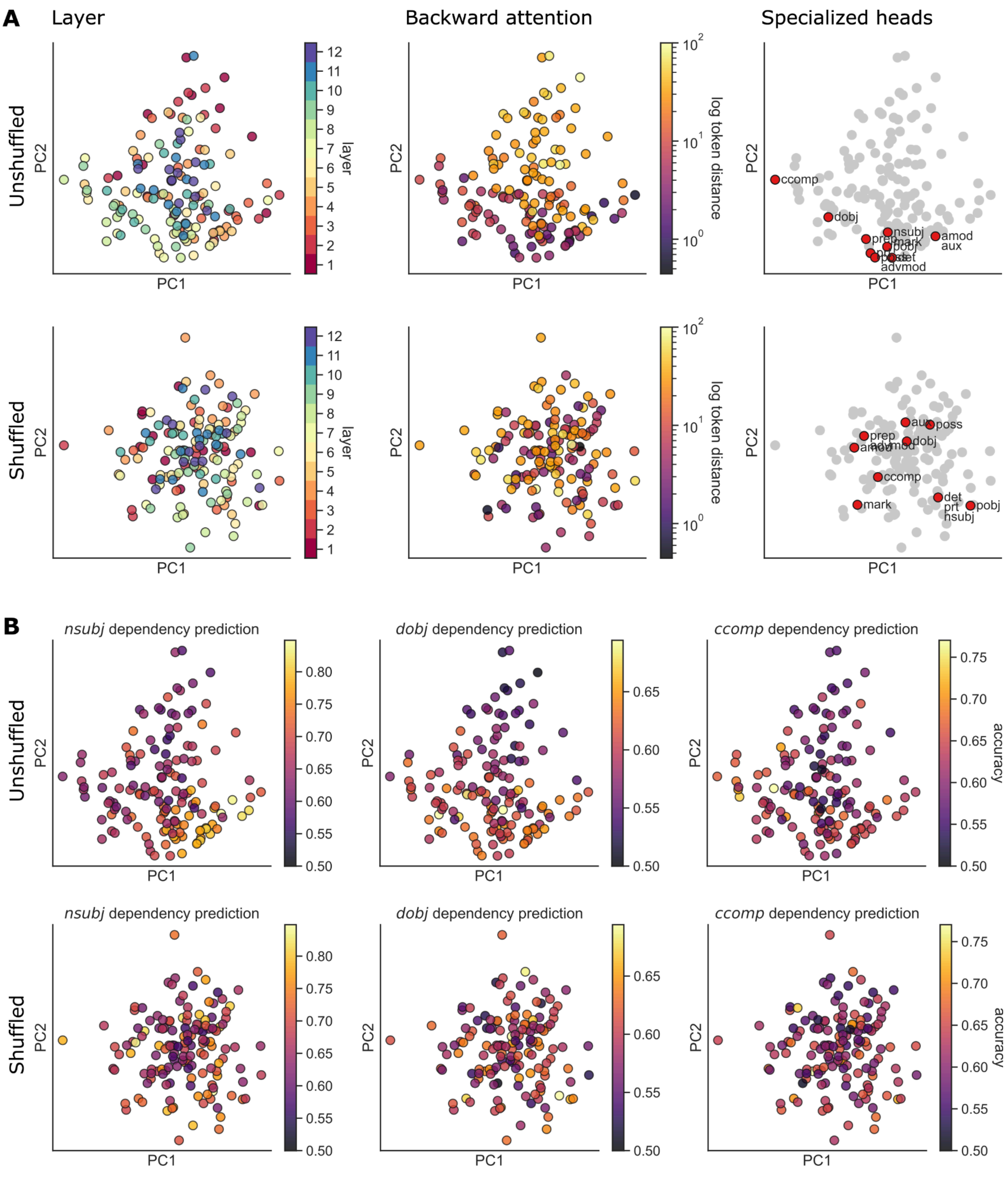

Figure S24. Control analysis in which we shuffle the coefficients assigned to transformation features by the encoding model across heads within each layer of BERT prior to running the analysis in Fig. 4 (i.e. immediately prior to computing the L2 norm in Fig. 4A). This perturbation effectively disrupts the functional grouping of transformation features into particular heads. (**A**) Heads are colored according to layer assignment, look-back distance (log-scale token distance), and functionally specialized heads (Clark et al., 2019) in the projection onto PCs 1 and 2. The original, unshuffled PCA solution (upper) is reproduced from Fig. 4 for comparison. In the

shuffled PCA solution (lower), there is no visible structure for layer assignment or look-back distance. Disrupting the grouping of transformation features into functionally-specialized heads abolishes the low-dimensional structure of headwise contributions to predicting brain activity. (**B**) Heads are colored according to their dependency prediction scores in the projection onto PCs 1 and 2. The unshuffled PCA solution (upper) is reproduced from Fig. S23 for the nsubj, dobj, and ccomp dependencies. Dependency decoding performance falls along visible gradients in this low-dimensional brain space. In the shuffled PCA solution (lower), there are no visible gradients in dependency decoding performance. Figure made using Matplotlib, seaborn, and Inkscape.

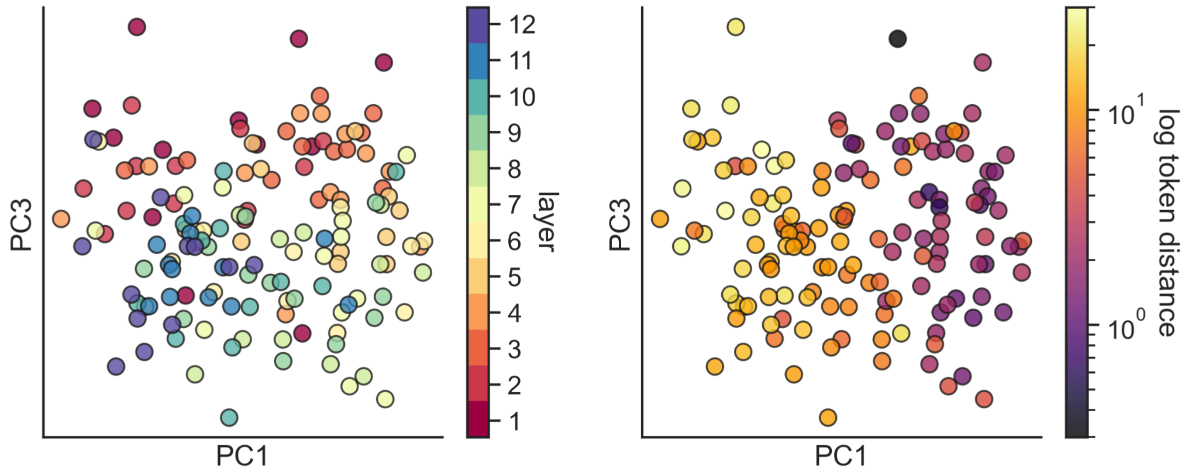

Figure S25. Headwise transformations in a low-dimensional brain space derived from GPT-2 (cf. Fig. 4). Following the same procedure used for BERT, we applied PCA to the weight vectors for transformation encoding models based on GPT-2 across language parcels, effectively projecting the transformation weights into a low-dimensional brain space. Each data point in the scatter plot corresponds to one of 144 heads in GPT-2. At left, heads are colored according to their layer in a reduced-dimension space of PC1 and PC3. A layer gradient is visible along PC3 ($r = .60$), similarly to PC1 and PC2 in BERT. At right, heads are colored according to their average backward attention distance in the story stimuli for PC1 and PC3 (look-back token distance is colored according to a log scale; color scale maximum = 50 tokens). Whereas look-back distance in BERT is most highly correlated with PC2, in GPT-2, PC1 reflects a strong gradient of look-back distance ($r = .77$). Figure made using Nilearn, Matplotlib and seaborn.

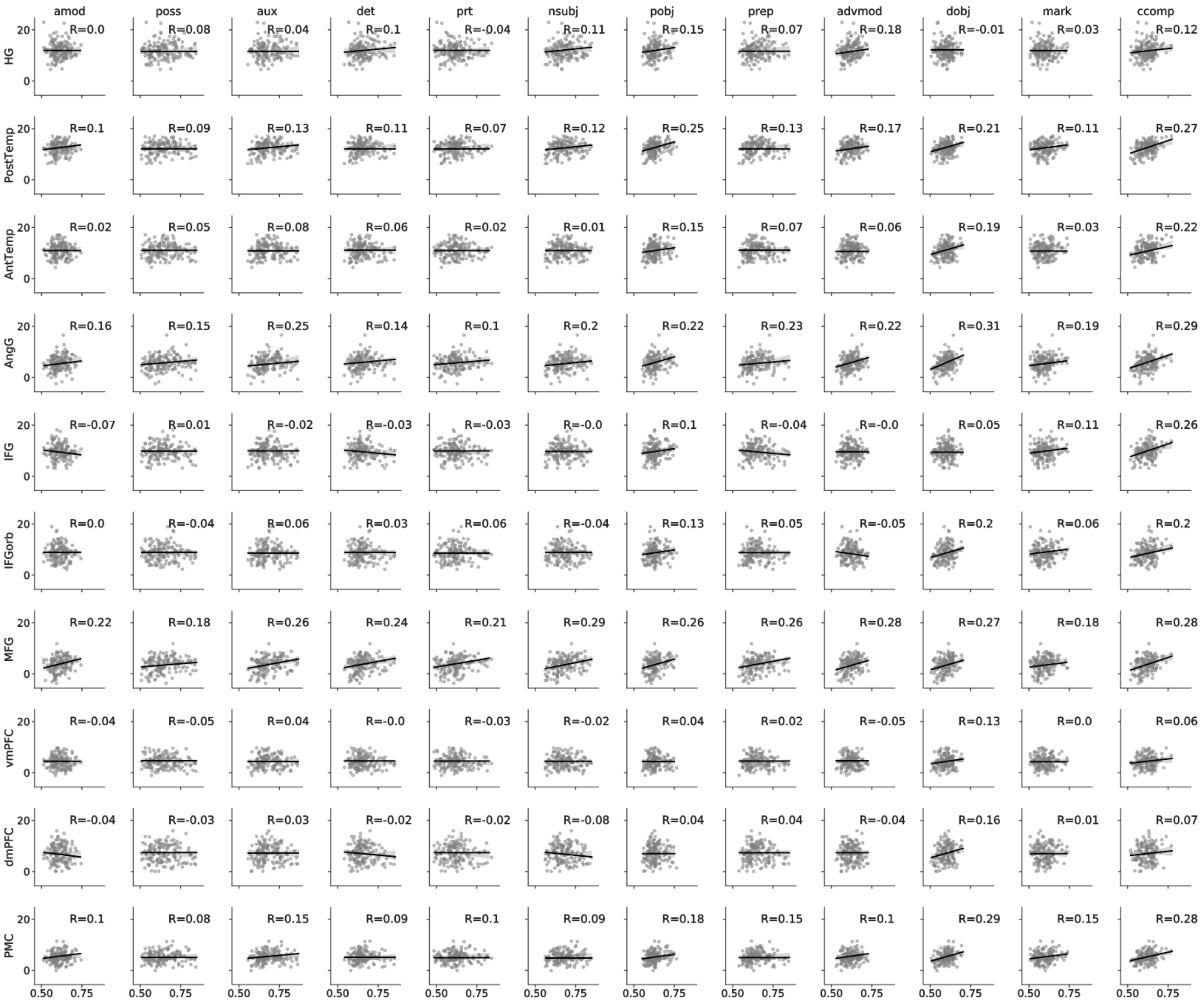

Figure S26. Correspondence between headwise brain prediction and dependency prediction scores for all dependencies (x-axis) and language ROIs (y-axis; cf. Fig. 5). Each point in a given scatter plot represents the dependency prediction (x-axis) and brain prediction (y-axis) scores for each of the 144 heads. Brain prediction scores reflect cross-validated encoding model performance evaluated in terms of the percent of a noise ceiling estimated using intersubject correlation. Dependency prediction scores reflect the classification accuracy of a cross-validated logistic regression model trained to predict the occurrence of a given linguistic dependency at each time point from the 64-dimensional transformation vector for a given attention head. Figure made using Nilearn, Matplotlib and seaborn.

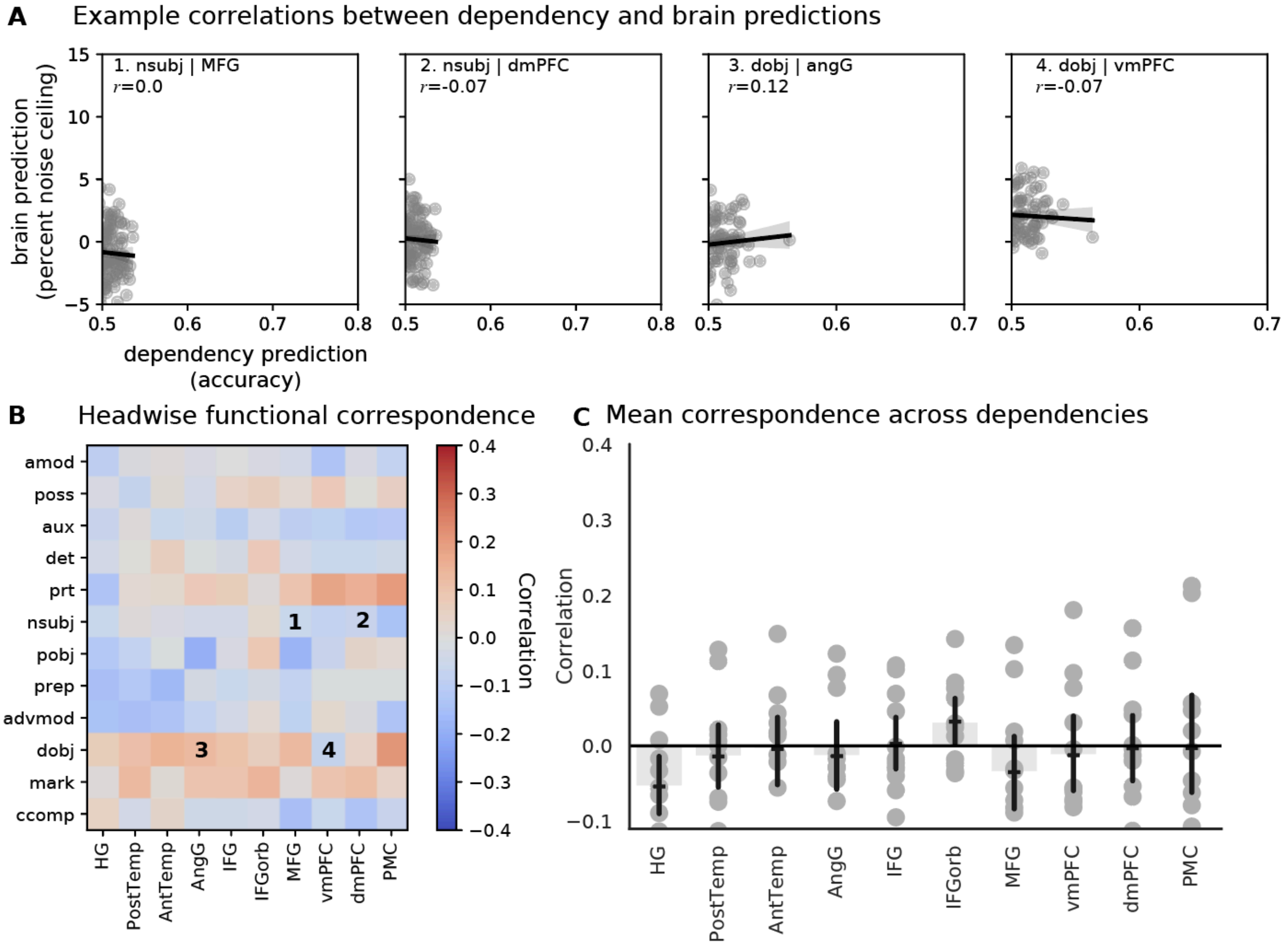

Figure S27. As a control analysis, we reevaluated headwise functional correspondence after shuffling the transformation features across heads within each layer (cf. Fig. 5). To do this, we first shuffled the transformation dimensions across heads within each layer, then recomputed both the brain prediction scores (the encoding model mapping transformations onto parcelwise brain activity) and the dependency prediction scores (the logistic regression model for predicting the occurrence of a given linguistic dependency) for each ROI and each linguistic dependency. We then segmented these shuffled transformations back into “pseudo-heads” and recomputed the functional correspondence of brain and dependency prediction scores across heads (cf. Fig. 5). This effectively abolishes the headwise structure of the transformations and any functional specialization therein. The shuffled transformations yields (**A**) lower brain and dependency prediction scores for the shuffled pseudo-heads and (**B**) reduces functional correspondence (none of the correlations were significant). (**C**) The 95% bootstrap confidence intervals for the mean functional correspondence across dependencies for each ROI cross zero, suggesting that there is no significant functional correspondence for any ROI. Figure made using Matplotlib, seaborn, and Inkscape.

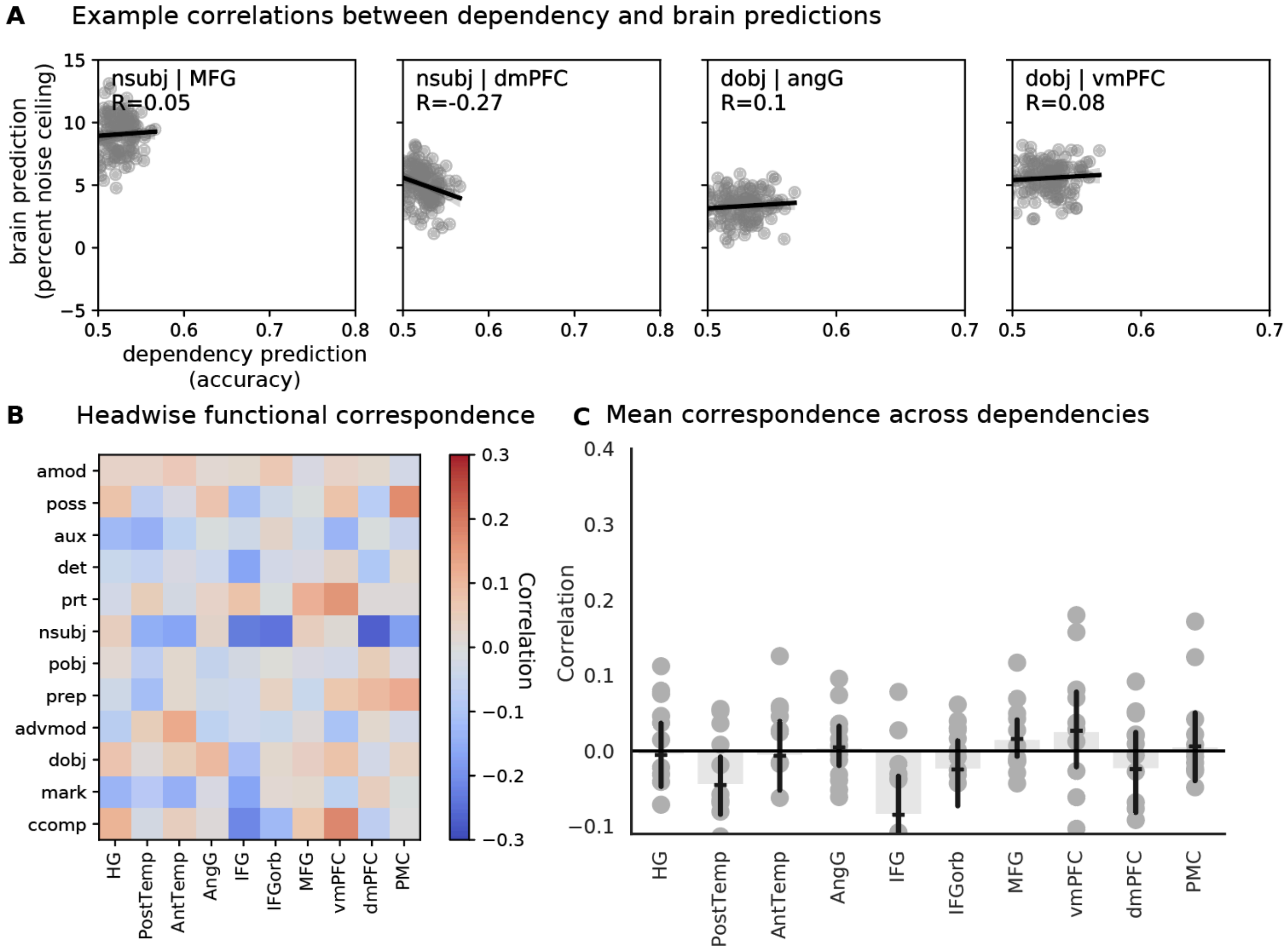

Figure S28. As a control analysis, we reevaluated headwise functional correspondence using an untrained, randomly-initialized instance of BERT (cf. Fig. 5). This is intended to be a strong control in that the model retains the same architecture as the trained model and receives the same text from our stimuli as input. The untrained BERT model, however, has not been trained to predict masked words across large corpora of text, and therefore the model's internal weights do not encode the statistical structure of real-world language. After extracting the transformation vectors from the untrained model based on our stimulus, we recomputed both the brain prediction scores (the encoding model mapping transformations onto parcelwise brain activity) and the dependency prediction scores (the logistic regression model for predicting the occurrence of a given linguistic dependency) for each ROI and each linguistic dependency. We then recomputed the functional correspondence of brain and dependency prediction scores across heads (cf. Fig. 5). The resulting correlations do not reveal any obvious structure across dependences or ROIs. The untrained model (**A**) yields lower brain and dependency prediction scores and (**B**) reduces functional correspondence (no significant correlations between brain and dependency prediction scores). (**C**) The 95% bootstrap confidence intervals for the mean functional correspondence across dependencies for each ROI approach or cross zero, suggesting that there is no significant functional correspondence for any ROI. Figure made using Matplotlib, seaborn, and Inkscape.

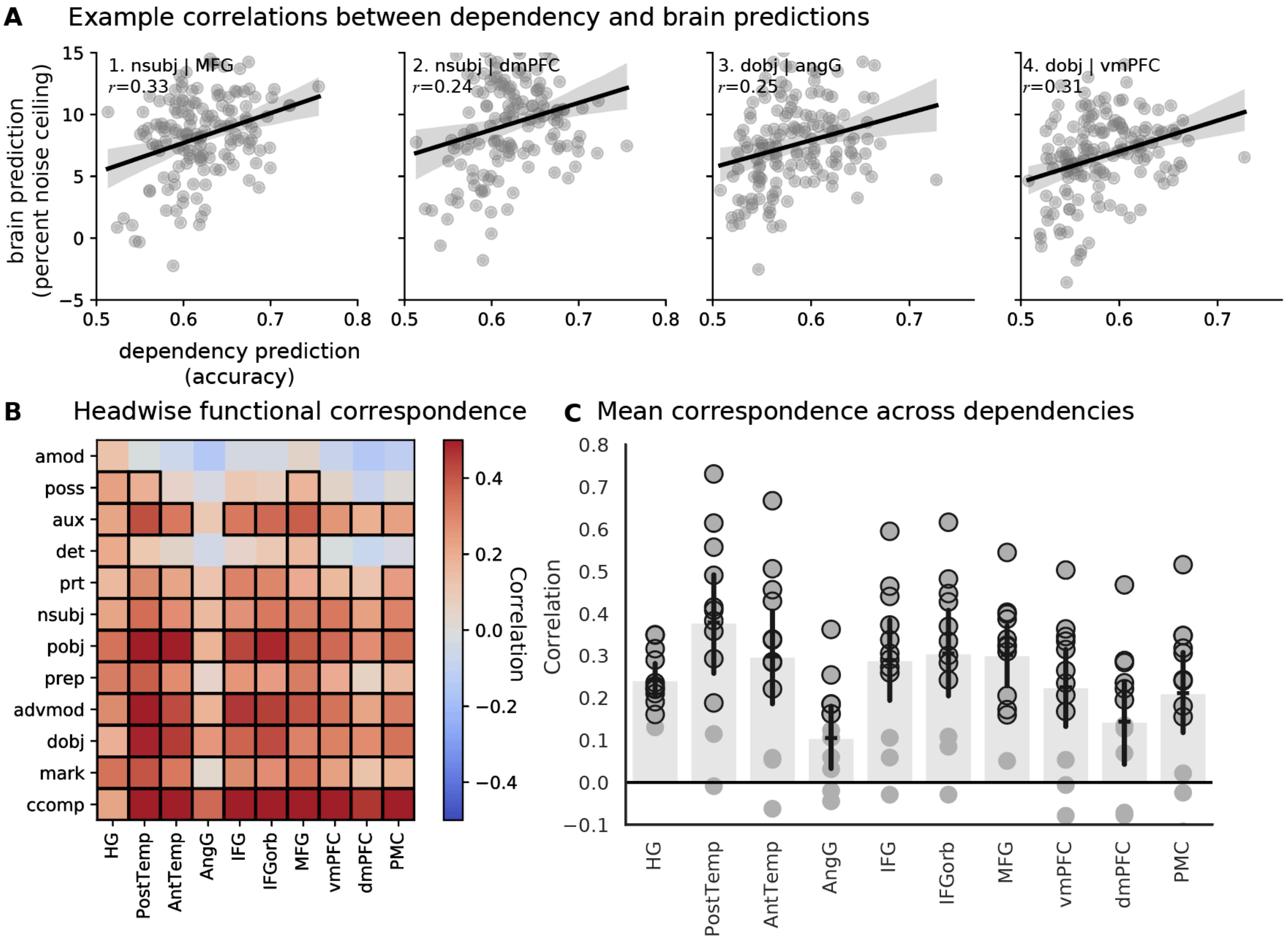

Figure S29. Correspondence between headwise brain and dependency predictions derived from GPT-2 (cf. Fig. 5). (**A**) Correlation between headwise brain prediction and dependency prediction scores for the same example ROIs and dependencies as Fig. 5. Each point in the scatter plot represents the dependency prediction (x-axis) and brain prediction (y-axis) scores for each of the 144 heads from GPT-2. (**B**) Correlation between headwise brain prediction and dependency prediction scores for each language ROI and syntactic dependency. Cells with black borders contain significant correlations as determined by a permutation test in which we shuffle assignments between headwise dependency prediction scores and brain prediction scores across heads (FDR controlled at $p < .05$). Labeled cells correspond to the example correlations in panel A. (**C**) We summarize the brain–dependency prediction correspondence for each ROI by averaging across syntactic dependencies (i.e. averaging each column of panel B). GPT-2 yields generally higher headwise correspondence than BERT (Fig. 5), but with less specificity across ROIs. Figure made using Matplotlib, seaborn, and Inkscape.

Comparison	HG	PostTemp	AntTemp	AngG	MFG	IFG	IFGorb	vmPFC	dmPFC	PMC
BERT transformations vs BERT embeddings	0.6620	0.0567	0.6700	0.9140	0.7887	0.7322	0.1517	1.0000	0.1867	0.0660
BERT transformations vs BERT transformation magnitudes	0.0114	0.0057	0.0025	0.0017	0.7000	0.0250	0.0025	0.0071	0.0017	0.0533
BERT transformations vs linguistic features	0.0017	0.0017	0.0025	0.0017	0.7000	0.0025	0.0025	0.0020	0.0017	0.0017
BERT transformations vs GloVe embeddings	0.0017	0.0017	0.0500	0.0017	0.7887	0.0025	1.0000	0.0020	0.0171	0.0017
BERT embeddings vs BERT transformation magnitudes	0.0017	0.0017	0.0025	0.0017	1.0000	0.0025	0.0025	0.0020	0.0017	0.0425
BERT embeddings vs linguistic features	0.0017	0.0017	0.0025	0.0017	0.7887	0.0025	0.0025	0.0020	0.0017	0.0017
BERT embedding vs GloVe embeddings	0.0017	0.0017	0.0140	0.0017	0.7887	0.0140	0.2256	0.0020	0.0017	0.0017
BERT transformation magnitudes vs linguistic features	0.0017	0.0017	0.6089	0.8087	0.7000	0.7322	0.1986	0.1212	0.7500	0.0017
BERT transformation magnitudes vs GloVe embeddings	0.0175	0.0488	0.0267	0.4814	0.8678	0.8540	0.2256	1.0000	0.1375	0.0017
Linguistic features vs GloVe embeddings	0.2633	0.0940	0.0663	0.8144	0.7000	0.6014	0.0200	0.0033	0.0017	0.0229

Table S1. Statistical significance of comparisons between BERT features (embeddings, transformations, transformation magnitudes), static GloVe embeddings, and classical linguistic features at each language ROI. For each comparison, two-tailed p-values reflect the statistical significance of the difference in encoding performance across subjects for a given ROI (permutation test; FDR controlled at $p < .05$).

Dependency	Mean performance across all heads	Top-performing head	Performance of top head
prep	0.654	1-11	0.846 (z = 3.0)
pobj	0.631	1-4	0.764 (z = 3.1)
det	0.658	3-1	0.852 (z = 3.2)
nsubj	0.682	3-1	0.849 (z = 2.6)
amod	0.613	3-10	0.745 (z = 3.0)
dobj	0.598	6-11	0.694 (z = 2.5)
advmod	0.622	1-11	0.728 (z = 2.6)
aux	0.686	2-2	0.863 (z = 2.6)
poss	0.646	6-1	0.844 (z = 2.5)
ccomp	0.620	5-11	0.770 (z = 3.4)
mark	0.597	7-10	0.740 (z = 3.0)
prt	0.619	3-1	0.841 (z = 3.1)

Table S2. Dependency prediction scores for transformations at top-performing attention heads. For each linguistic dependency, we used logistic regression with five-fold cross-validation to predict the binary occurrence of the dependency across TRs from the 64-dimensional transformation vectors separately at each attention head (see "Decoding dependency relations" in the Methods section). Given that some dependencies only occur at relatively few TRs (i.e. unbalanced class frequencies), decoding performance was evaluated using balanced classification accuracy (theoretical chance = 0.5). We first report the mean balanced accuracy across all 144 attention heads (mean accuracy = 0.636 across all dependencies). For each dependency, we then report the head with the highest decoding performance (e.g. "1-11" indicates head 11 in layer 1 of BERT). For the top-performing head, we report the balanced accuracy and the z-score for this accuracy relative to the distribution of accuracies across all heads.

ROI	Embedding Entropy	Transformation Entropy	Difference	p-value
PostTemp	1.2130171	0.68240984	5.126	0.00016667
AntTemp	0.967746731	0.3861663	6.664	0.00016667
AngG	0.635415239	0.38440445	2.635	0.0122
IFG	0.849934771	0.37969937	4.214	0.00342857
MFG	0.642895319	0.39010882	3.005	0.0122
IFGorb	0.710395586	0.3873914	3.356	0.0122
vmPFC	0.819666132	0.41612847	3.975	0.00016667
dmPFC	0.767067125	0.37410996	5.21	0.00016667
PMC	1.005453546	0.6119654	3.604	0.00016667
HG	1.067141906	0.51129539	4.432	0.00016667

Table S3. Mean entropy of the distribution of encoding performance across layers for embeddings and transformations at each language ROIs. Statistical significance for the difference between entropy for embeddings and transformations was evaluated by bootstrap resampling the differences (FDR controlled at $p < .05$).

Part-of-speech	Description	Examples
PRON	pronoun	she, somebody, mine
VERB	verb	run, eating
NOUN	noun	girl, tree, air
DET	determiner	a, the, this
AUX	auxiliary	is, will, should
ADP	adposition	in, to,during
ADV	adverb	well, tomorrow, very
CCONJ	coordinating conjunction	and, or, but
ADJ	adjective	big, green, first
PART*	particle	’s, not
PROPN	proper noun	Mary, John, London
SCONJ	subordinating conjunction	if, while
NUM	numeral	one, two, three
INTJ	interjection	psst, bravo

Table S4. Descriptions and examples of the parts-of-speech. Examples are excerpted from https://universaldependencies.org/u/pos/. Phrasal verb particles, e.g. [give] in, are not included; they are tagged as ADP or ADV.

Dependency	Description	Example
amod	adjectival modifier	Sam eats [large]$_{amod}$ hot [dogs]$_{head}$.
poss	possessive nominal modifier	[Marie]$_{poss}$ ’s [book]$_{head}$
aux	auxiliary	He [should]$_{aux}$ [leave]$_{head}$.
det	determiner	[Which]$_{det}$ [book]$_{head}$ do you prefer?
prt	phrasal verb particle	They [shut]$_{head}$ [down]$_{prt}$ the station.
nsubj	nominal subject	The [car]$_{nsubj}$ is [red]$_{head}$.
pobj*	object of preposition	We went [to]$_{head}$ the grocery [store]$_{pobj}$.
prep*	preposition	We [went]$_{head}$ [to]$_{prep}$ the grocery store.
advmod	adverbial modifier	[Genetically]$_{advmod}$ [modified]$_{head}$ food
dobj	direct object	She [gave]$_{head}$ me a [raise]$_{dobj}$.
mark	subordinate clause marker	He says [that]$_{mark}$ you [like]$_{head}$ to swim.
ccomp	clausal complement	He [says]$_{head}$ that you [like]$_{ccomp}$ to swim.

Table S5. Descriptions and examples of the dependency relations in Fig. 5. The labels are part of ClearNLP dependency labels (https://github.com/clir/clearnlp-guidelines/blob/master/md/specifications/dependency_labels.md). Examples are adapted from https://universaldependencies.org/u/dep/all.html. Note that poss, prt, and dobj in the table correspond to nmod:poss, compound:prt, and obj in the universal dependencies, respectively; pobj and prep are not part of the universal dependencies.

**Dependency**	**“black”** (~/534)	**“slumlord”** (~/619)
prep	121	214
pobj	109	195
det	117	206
nsubj	245	392
amod	43	66
dobj	95	153
advmod	122	177
aux	99	150
poss	35	45
ccomp	46	61
mark	19	35
prt	13	29

Table S6. Number of TRs in which linguistic dependency occurs in the two story stimuli. The column headers indicate the total number of TRs for that stimulus.
