# Supplementary material for: Shared functional specialization in transformer-based language models and the human brain
Source: Nat Commun. 2024 Jun 29;15:5523. doi: 10.1038/s41467-024-49173-5 (PMC11217339; doi:10.1038/s41467-024-49173-5)
Supplement: Supplementary file 3 — Reporting Summary [file 41467_2024_49173_MOESM3_ESM.pdf]

nature portfolio

Corresponding author(s): Samuel A. Nastase

Last updated by author(s): Apr 25, 2024

Reporting Summary

Nature Portfolio wishes to improve the reproducibility of the work that we publish. This form provides structure for consistency and transparency in reporting. For further information on Nature Portfolio policies, see our Editorial Policies and the Editorial Policy Checklist.

Statistics

For all statistical analyses, confirm that the following items are present in the figure legend, table legend, main text, or Methods section.

n/a	Confirmed	
☐	☒	The exact sample size (n) for each experimental group/condition, given as a discrete number and unit of measurement
☐	☒	A statement on whether measurements were taken from distinct samples or whether the same sample was measured repeatedly
☐	☒	The statistical test(s) used AND whether they are one- or two-sided<br>*Only common tests should be described solely by name; describe more complex techniques in the Methods section.*
☐	☒	A description of all covariates tested
☐	☒	A description of any assumptions or corrections, such as tests of normality and adjustment for multiple comparisons
☐	☒	A full description of the statistical parameters including central tendency (e.g. means) or other basic estimates (e.g. regression coefficient) AND variation (e.g. standard deviation) or associated estimates of uncertainty (e.g. confidence intervals)
☐	☒	For null hypothesis testing, the test statistic (e.g. F, t, r) with confidence intervals, effect sizes, degrees of freedom and P value noted<br>*Give P values as exact values whenever suitable.*
☒	☐	For Bayesian analysis, information on the choice of priors and Markov chain Monte Carlo settings
☒	☐	For hierarchical and complex designs, identification of the appropriate level for tests and full reporting of outcomes
☐	☒	Estimates of effect sizes (e.g. Cohen's d, Pearson's r), indicating how they were calculated

Our web collection on statistics for biologists contains articles on many of the points above.

Software and code

Policy information about availability of computer code

Data collection	The publicly available, archival fMRI used in this study were acquired using proprietary software on a Siemens MRI scanner. The archival data were shared in keeping with best practices in transparency and reproducibility (version control using DataLad, data standardization with BIDS). Stimuli were presented using PsychoPy 2. The collection of these archival data are described in full detail in the following data descriptor paper: https://doi.org/10.1038/s41597-021-01033-3. Language models were accessed using the Hugging Face environment: https://huggingface.co/.
Data analysis	Data were preprocessed using fMRIPrep 20.0.5 and AFNI 19.3.0, then further analyzed using open-source Python v3 code; all code are shared via GitHub: https://github.com/tsumers/bert-brains.

For manuscripts utilizing custom algorithms or software that are central to the research but not yet described in published literature, software must be made available to editors and reviewers. We strongly encourage code deposition in a community repository (e.g. GitHub). See the Nature Portfolio guidelines for submitting code & software for further information.

Data

Policy information about availability of data

All manuscripts must include a data availability statement. This statement should provide the following information, where applicable:

- Accession codes, unique identifiers, or web links for publicly available datasets
- A description of any restrictions on data availability
- For clinical datasets or third party data, please ensure that the statement adheres to our policy

The MRI data used in this study are openly available as part of the "Narratives" dataset (Nastase et al., 2021), publicly available via the OpenNeuro repository at https://doi.org/10.18112/openneuro.ds002345.v1.1.4, and via DataLad at https://datasets.datalad.org/?dir=/labs/hasson/narratives. The Schaefer atlas was obtained from the associated GitHub repository: https://github.com/ThomasYeoLab/CBIG/tree/master/stable_projects/brain_parcellation/Schaefer2018_LocalGlobal. The language ROIs were obtained from Fedorenko and colleagues: https://evlab.mit.edu/funcloc/. The Harvard-Oxford atlas was obtained from FSL: https://fsl.fmrib.ox.ac.uk/fsl/fslwiki/Atlases. Source data are provided with this paper.

Research involving human participants, their data, or biological material

Policy information about studies with human participants or human data. See also policy information about sex, gender (identity/presentation), and sexual orientation and race, ethnicity and racism.

Reporting on sex and gender	None of our hypotheses vary by sex or gender, and we do not analyze sex or gender variables. In keeping with the literature and BIDS standard, we use the term "sex" in demographic information, but formulate this as "reported sex" to remain sensitive to different gender identities.
Reporting on race, ethnicity, or other socially relevant groupings	We do not report race, ethnicity, or other social variables, as these variables are not related to any of our hypotheses or analyses.
Population characteristics	In keeping with the BIDS standard, we report "age" and "reported sex". However, these variables are not related to any of our hypotheses or analyses.
Recruitment	Participants were recruited from the Princeton University student body as well as non-university-affiliated members of the broader community in Princeton, NJ
Ethics oversight	All participants provided informed, written consent prior to data collection in accordance with experimental procedures approved by Princeton University Institutional Review Board

Note that full information on the approval of the study protocol must also be provided in the manuscript.

Field-specific reporting

Please select the one below that is the best fit for your research. If you are not sure, read the appropriate sections before making your selection.

☒ Life sciences ☐ Behavioural & social sciences ☐ Ecological, evolutionary & environmental sciences

For a reference copy of the document with all sections, see nature.com/documents/nr-reporting-summary-flat.pdf

Life sciences study design

All studies must disclose on these points even when the disclosure is negative.

Sample size	Publicly available data were used with a sample size of N = 63 subjects—that is, all available subjects for two relatively long stories from the archival dataset. This dramatically exceeds sample sizes used in several high-profile examples from the surrounding literature (e.g. Huth et al., 2016; Schrimpf et al., 2021).
Data exclusions	No data were excluded.
Replication	The core analyses were performed in two separate samples of subjects listening to two separate story stimuli. We observed qualitatively similar results in both story stimuli.
Randomization	No subject- or group-level manipulations requiring randomization were performed. Subjects were randomly sampled from the population of students and community members in the vicinity of Princeton University as described in Nastase et al., 2021.
Blinding	No subject- or group-level manipulations requiring blinding were performed. Subjects were blind to the modeling procedures and goals of the current experiment.

Reporting for specific materials, systems and methods

We require information from authors about some types of materials, experimental systems and methods used in many studies. Here, indicate whether each material, system or method listed is relevant to your study. If you are not sure if a list item applies to your research, read the appropriate section before selecting a response.

Materials & experimental systems

n/a	Involved in the study
☒	☐ Antibodies
☒	☐ Eukaryotic cell lines
☒	☐ Palaeontology and archaeology
☒	☐ Animals and other organisms
☒	☐ Clinical data
☒	☐ Dual use research of concern
☒	☐ Plants

Methods

n/a	Involved in the study
☒	☐ ChIP-seq
☒	☐ Flow cytometry
☐	☒ MRI-based neuroimaging

Plants

Seed stocks: n/a

Novel plant genotypes: n/a

Authentication: n/a

Magnetic resonance imaging

Experimental design

Design type: Naturalistic story-listening design

Design specifications: No blocks or trials were used. Subjects listened to naturalistic spoken stories that were each roughly 13 minutes long.

Behavioral performance measures: Story comprehension was behaviorally assessed using questionnaires, but these were not used in the current study.

Acquisition

Imaging type(s): Functional, structural

Field strength: 3 Tesla

Sequence & imaging parameters: Functional images were acquired in an interleaved fashion using gradient-echo EPI with a multiband (simultaneous multi-slice; SMS) acceleration factor of 4 using blipped CAIPIRINHA and no in-plane acceleration: TR/TE 1500/39 ms, flip angle = 50°, bandwidth=1240Hz/Px, in-plane resolution=2.0×2.0mm, slice thickness 2.0mm, matrix size=96×96, FoV= 192×192mm, 60 axial slices with full brain coverage and no gap, anterior–posterior phase encoding, 6/8 partial Fourier, no prescan normalization, fat suppression, three dummy scans. T1-weighted structural images were acquired using a high-resolution single-shot MPRAGE sequence with an in-plane acceleration factor of 2 using GRAPPA: TR/TE/TI= 2530/2.67/1200ms, flip angle=7°, bandwidth=200Hz/Px, in-plane resolution 1.0×1.0 mm, slice thickness 1.0mm, matrix size=256×256, FoV=176×256×256mm, 176 sagittal slices, ascending acquisition, no fat suppression, 5minutes 52seconds total acquisition time.

Area of acquisition: Head

Diffusion MRI: ☐ Used ☒ Not used

Preprocessing

Preprocessing software: Anatomical images were de-faced using the automated de-facing software pydeface 2.0.0134 prior to further processing (using the run_pydeface.py script in the code/ directory). MRI data were subsequently preprocessed using fMRIPrep 20.0.5135,136 (RRID:SCR_016216; using the run_fmriprep.sh script in the code/ directory). FMRIPrep is a containerized, automated tool based on Nipype 1.4.2137,138 (RRID:SCR_002502) that adaptively adjusts to idiosyncrasies of the dataset (as captured by the metadata) to apply the best-in-breed preprocessing workflow. Many internal operations of fMRIPrep functional processing workflow use Nilearn 0.6.2139 (RRID:SCR_001362). For more details of the pipeline, see the section corresponding to workflows in fMRIPrep's documentation. The containerized fMRIPrep software was deployed using

Singularity 3.5.2-1.1.sdl7140. The fMRIPrep Singularity image can be built from Docker Hub (https://hub.docker.com/r/poldracklab/fmriprep/; e.g. singularity build fmriprep-20.0.5.simg docker://poldracklab/fmriprep:20.0.5). The fMRIPrep outputs and visualization can be found in the fmriprep/ directory in derivatives/ available via the DataLad release. The fMRIPrep workflow produces two principal outputs: (a) the functional time series data in one more output space (e.g. MNI space), and (b) a collection of confound variables for each functional scan. In the following, we describe fMRIPrep's anatomical and functional workflows, as well as subsequent spatial smoothing and confound regression implemented in AFNI 19.3.0141,142 (RRID:SCR_005927).

The anatomical MRI T1-weighted (T1w) images were corrected for intensity non-uniformity with N4BiasFieldCorrection143, distributed with ANTs 2.2.0144 (RRID:SCR_004757), and used as T1w-reference throughout the workflow. The T1w-reference was then skull-stripped with a Nipype implementation of the antsBrainExtraction.sh (from ANTs) using the OASIS30ANTs as the target template. Brain tissue segmentation of cerebrospinal fluid (CSF), white-matter (WM), and gray-matter (GM) was performed on the brain-extracted T1w using fast145 (FSL 5.0.9; RRID:SCR_002823). Brain surfaces were reconstructed using recon-all146,147 (FreeSurfer 6.0.1; RRID:SCR_001847), and the brain mask estimated previously was refined with a custom variation of the method to reconcile ANTs-derived and FreeSurfer-derived segmentations of the cortical gray-matter from Mindboggle148 (RRID:SCR_002438). Volume-based spatial normalization to two commonly-used standard spaces (MNI152NLin2009cAsym, MNI152NLin6Asym) was performed through nonlinear registration with antsRegistration (ANTs 2.2.0) using brain-extracted versions of both T1w reference and the T1w template. The following two volumetric templates were selected for spatial normalization and deployed using TemplateFlow149: (a) ICBM 152 Nonlinear Asymmetrical Template Version 2009c150 (RRID:SCR_008796; TemplateFlow ID: MNI152NLin2009cAsym), and (b) FSL's MNI ICBM 152 Non-linear 6th Generation Asymmetric Average Brain Stereotaxic Registration Model151 (RRID:SCR_002823; TemplateFlow ID: MNI152NLin6Asym). Surface-based normalization based on nonlinear registration of sulcal curvature was applied using the following three surface templates152 (FreeSurfer reconstruction nomenclature): fsaverage, fsaverage6, fsaverage5.

The functional MRI data were preprocessed in the following way. First, a reference volume and its skull-stripped version were generated using a custom methodology of fMRIPrep. A deformation field to correct for susceptibility distortions was estimated using fMRIPrep's fieldmap-less approach. The deformation field results from co-registering the BOLD reference to the same-subject T1w-reference with its intensity inverted153,154. Registration was performed with antsRegistration (ANTs 2.2.0), and the process was regularized by constraining deformation to be nonzero only along the phase-encoding direction, and modulated with an average fieldmap template155. Based on the estimated susceptibility distortion, a corrected EPI reference was calculated for more accurate co-registration with the anatomical reference. The BOLD reference was then co-registered to the T1w reference using bbregister (FreeSurfer 6.0.1), which implements boundary-based registration156. Co-registration was configured with six degrees of freedom. Head-motion parameters with respect to the BOLD reference (transformation matrices, and six corresponding rotation and translation parameters) are estimated before any spatiotemporal filtering using mcflirt (FSL 5.0.9)157,158,159. BOLD runs were slice-time corrected using 3dTshift from AFNI 20160207160. The BOLD time-series were resampled onto the following surfaces: fsaverage, fsaverage6, fsaverage5. The BOLD time-series (including slice-timing correction when applied) were resampled onto their original, native space by applying a single, composite transform to correct for head-motion and susceptibility distortions. These resampled BOLD time-series are referred to as preprocessed BOLD in original space, or just preprocessed BOLD. The BOLD time-series were resampled into two volumetric standard spaces, correspondingly generating the following spatially-normalized, preprocessed BOLD runs: MNI152NLin2009cAsym, MNI152NLin6Asym. A reference volume and its skull-stripped version were first generated using a custom methodology of fMRIPrep. All resamplings were performed with a single interpolation step by composing all the pertinent transformations (i.e. head-motion transform matrices, susceptibility distortion correction, and co-registrations to anatomical and output spaces). Gridded (volumetric) resamplings were performed using antsApplyTransforms (ANTs 2.2.0), configured with Lanczos interpolation to minimize the smoothing effects of other kernels161. Non-gridded (surface) resamplings were performed using mri_vol2surf (FreeSurfer 6.0.1).

Normalization	Functional images were normalized to MNI space using ANTs.
Normalization template	MNI152NLin2009cAsym
Noise and artifact removal	Several confounding time-series were calculated based on the preprocessed BOLD: framewise displacement (FD), DVARS, and three region-wise global signals. FD and DVARS are calculated for each functional run, both using their implementations in Nipype162. The three global signals are extracted within the CSF, the WM, and the whole-brain masks. Additionally, a set of physiological regressors were extracted to allow for component-based noise correction (CompCor)163. Principal components are estimated after high-pass filtering the preprocessed BOLD time-series (using a discrete cosine filter with 128s cut-off) for the two CompCor variants: temporal (tCompCor) and anatomical (aCompCor). The tCompCor components are then calculated from the top 5% variable voxels within a mask covering the subcortical regions. This subcortical mask is obtained by heavily eroding the brain mask, which ensures it does not include cortical GM regions. For aCompCor, components are calculated within the intersection of the aforementioned mask and the union of CSF and WM masks calculated in T1w space, after their projection to the native space of each functional run (using the inverse BOLD-to-T1w transformation). Components are also calculated separately within the WM and CSF masks. For each CompCor decomposition, the k components with the largest singular values are retained, such that the retained components' time series are sufficient to explain 50 percent of variance across the nuisance mask (CSF, WM, combined, or temporal). The remaining components are dropped from consideration. The head-motion estimates calculated in the correction step were also placed within the corresponding confounds file. The confound time series derived from head motion estimates and global signals were expanded with the inclusion of temporal derivatives and quadratic terms for each164.
Volume censoring	None

Statistical modeling & inference

Model type and settings	Mass univariate encoding model using regularized ridge regression.
Effect(s) tested	Linguistic encoding model performance was assessed in terms of the correlation between predicted and actual parcel time

series using cross-validation.

Specify type of analysis: ☐ Whole brain ☐ ROI-based ☒ Both

Anatomical location(s): The whole-brain 1000-area cortical parcellation from Schaefer et al., 2018, was used.

Statistic type for inference (See Eklund et al. 2016): No cluster-wise statistics were reported; statistics were evaluated per parcel and corrected for multiple tests using FDR.

Correction: All statistics were corrected for multiple tests by controlling the false discovery rate (FDR) at p < .05

Models & analysis

n/a	Involved in the study
☒	☐ Functional and/or effective connectivity
☒	☐ Graph analysis
☐	☒ Multivariate modeling or predictive analysis

Multivariate modeling and predictive analysis: Linguistic features were used to predict parcelwise response time series using ridge regression with cross-validation.
